# Targeting c-Myc-p300-CARM1 complex induces ferroptosis and reduces CD8^+^ T cell exhaustion in esophageal squamous cell carcinoma

**DOI:** 10.7150/ijbs.114575

**Published:** 2026-01-01

**Authors:** Yuhao Wang, Yang Li, Guanzhu Ren, Jin Zhou, Wangtianjiao Chen, Kai Zhang, Xiao Yu, Yin Yin, Ji Cong, Lei Ma, Xinyao Zheng, Yahui Zhao, Zhihua Liu

**Affiliations:** 1 State Key Laboratory of Molecular Oncology, National Cancer Center, National Clinical Research Center for Cancer, Cancer Hospital, Chinese Academy of Medical Sciences and Peking Union Medical College, Beijing 100021, China.; 2 Division of Liver Surgery, Department of General Surgery and Laboratory of Liver Surgery, West China Hospital, Sichuan University, China.

## Abstract

Amplification and high expression of the *c-Myc* gene promote the proliferation and metastasis of cancer, contributing to treatment resistance and poor prognosis. In this study, we analyzed whole genome sequencing data from 663 pairs of esophageal squamous cell carcinoma (ESCC) tumors and matched adjacent noncancerous esophagus tissues. The analysis revealed that *c-Myc* had an amplification rate of 16.4%, and its high expression was significantly associated with tumor metastasis, chemotherapy resistance, and poor prognosis of the patients. Drugs that can inhibit the oncogenic function of *c-Myc* currently have limited effects. Therefore, we screened for inhibitors that can sensitize *c-Myc* inhibitors. We found that SGC2085, a *CARM1* inhibitor, enhanced the efficacy of MYCi975, a *c-Myc* inhibitor. This combination disrupts the transcriptional c-Myc-p300-CARM1 (CPC) complex by R371 with R372 in c-Myc and E75 with Y153 in CARM1. Additionally, the combination promotes the accumulation of arachidonic acid, which in turn induces ferroptosis. Furthermore, the combination of SGC2085 and MYCi975 significantly increased B cells, CD8^+^T cells infiltration and decreased the level of CD8^+^ T cell exhaustion, neutrophils, and MDSC. These findings revealed that SGC2085 and MYCi975 could disrupt the transcriptional complex CPC, affect metabolic pathways, and reprogram the immune microenvironment. This study provides a potential therapeutic strategy for ESCC patients.

## Introduction

Esophageal cancer ranks eleventh in incidence and seventh in mortality worldwide 1, with an overall 5-year survival rate of 35% 2. Approximately 50% of the cases occur in East and Central Asia, especially in China, where esophageal squamous cell carcinoma (ESCC) is the predominant histological type. Common treatments include surgery, radiotherapy, chemotherapy, and immunotherapy 3. Whole-genome sequencing of ESCC clinical tumor tissue pairs in our group and many others has revealed that *c-Myc* has a high frequency of amplification in ESCC 4, 5, highlighting the potential impact of targeting *c-Myc* to improve treatment outcomes.

*C-Myc* encodes a family of transcription factors and is one of the most activated oncoproteins in human tumors, regulating numerous biological processes that directly or indirectly affect the expression of thousands of genes. Most tumors exhibit aberrant *MYC* activation and a high amplification ratio. The aberrant expression of *c-Myc* is significantly associated with altered tumor metabolism and immune microenvironment 6. Based on the main mechanism of action of MYC, patients with MYC-driven tumors can be further classified according to their phenotypes and thus receive different treatments. For instance, patients with MYC-amplified tumors can be treated directly with MYC inhibitors 7. Patients with enriched synthetic lethal MYC targets can be treated with drugs targeting specific gene products 8. Patients with immune cell-suppressed tumors can benefit from MYC-directed immunotherapy 9. Patients with MYC-induced metabolic dysregulation can be treated with drugs targeting metabolic pathways 10, 11. Therefore, elucidating the mechanisms underlying MYC carcinogenesis enables the development of selective and targeted therapies.

Currently, research on *c-Myc* inhibitors is still in its early stages. A recent Phase I clinical trial was completed for an Omomyc-based drug, OMO-103 12. MYC-MAX dimers normally bind to DNA to regulate protein expression, whereas Omomyc binding to *MYC* separates the natural transcription factors from their binding DNA. The Omomyc-Omomyc and Omomyc-MAX dimers simultaneously bind to DNA without altering transcription, thereby blocking the function of the true MYC-MAX dimer. Some drugs block the formation of MYC-MAX dimers and exert anti-tumor effects. Our findings showed that c-Myc forms a transcriptional complex with p300 and CARM1, which regulates arachidonic acid metabolism and *de novo* pyrimidine biosynthesis.

*CARM1*, also known as *PRMT4*, a protein arginine methyltransferase 13, is an epigenetic target with clinical potential to methylate a wide range of proteins 14, 15. Nine members of the *PRMT* family (*PRMT1-9*) have been identified, which can transfer the methyl group of S-adenosylmethionine to the guanidinium nitrogen of the protein arginine side chains to generate methylated arginine 16. *PRMTs* regulate arginine methylation in three different forms: monomethylarginine (MMA), asymmetric dimethylarginine (ADMA), and symmetric dimethylarginine (SDMA) 17. Based on these forms, PRMTs are classified into the following three types 18-20: Type I, consists mainly of *PRMT1*, *PRMT2*, *PRMT3*, *PRMT4*, *PRMT6*, and *PRMT8*, catalyzes ADMA formation from the substrate; type II, consisting of *PRMT5* and *PRMT9*, catalyzes SDMA formation from the substrate; and type III, consisting of *PRMT7*, catalyzes MMA. Protein methylation catalyzed by PRMTs regulates important cellular processes such as DNA repair, cell cycle progression, transcriptional regulation, and RNA splicing, highlighting *PRMTs* as potential oncogenes 21-25.

This study aimed to explore the mechanism of *c-Myc* in ESCC and identify a therapeutic strategy for *c-Myc* drug combinations by investigating the related mechanisms of the downstream pathways. We knocked down *c-Myc* and found that SGC2085 was more effective in the low *c-Myc* expression group through drug screening. The immune microenvironment and metabolic pathways were reprogrammed by SGC2085 and MYCi975. This study provides a potential therapeutic strategy for ESCC patients.

## Results

### Screening of drug combination regimens targeting *c-Myc*

**Figure 1:**
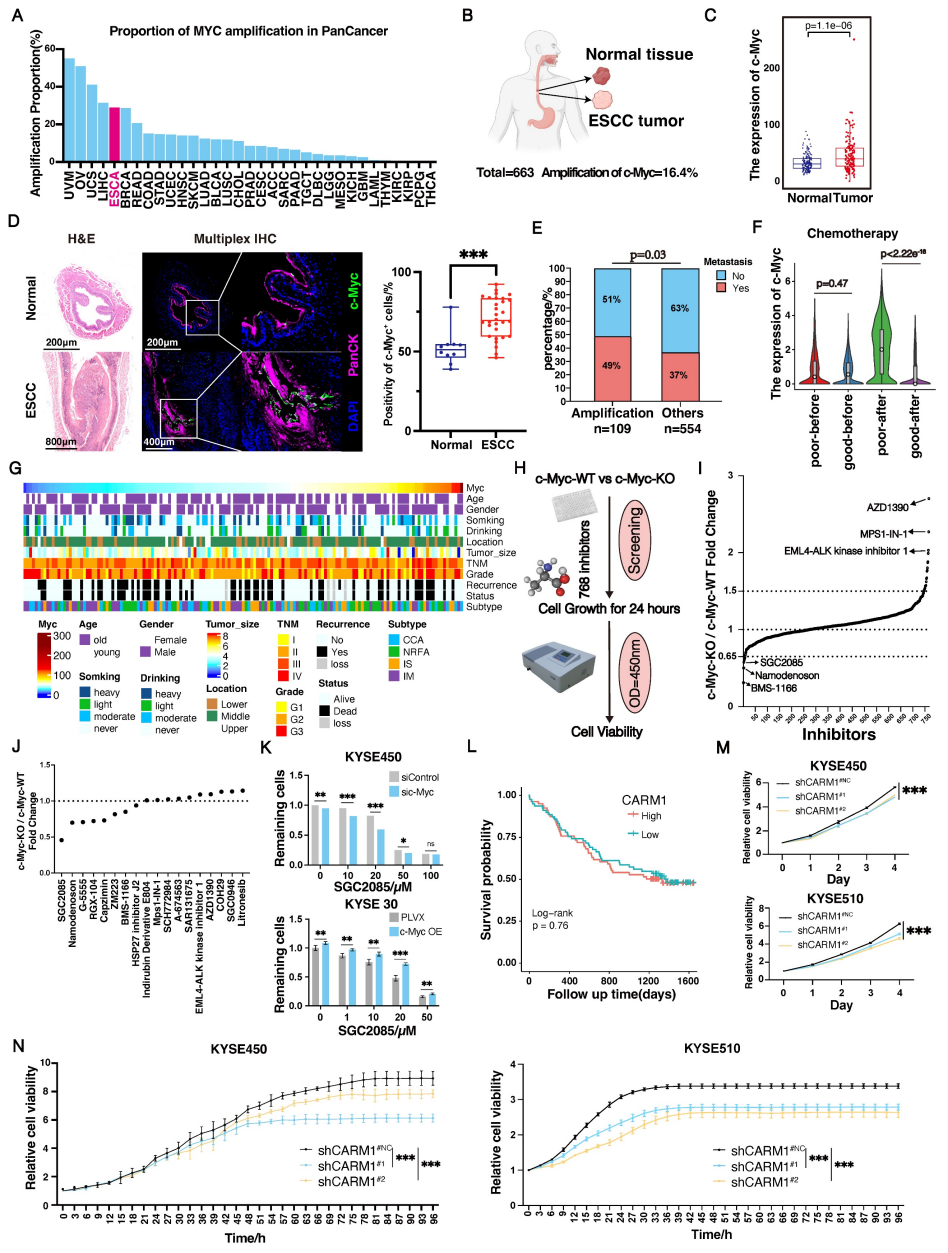
** Screening of drug combination regimens targeting c-Myc.** (A-B) c-Myc amplification rate in Pan cancer (A) and our ESCC cohort (n=663) (B). (C) The expression of *c-Myc* between normal tissue and tumor (n=663). (D) Representative mIHC image and matched false hematoxylin and eosin images showing the expression of indicated marker genes in esophageal tissue from normal mice and esophageal squamous cell carcinoma mice induced by 4NQO (n=7). (E) The differences of metastasis state between* c-Myc* amplification and other patients. (F) The relationship between *c-Myc* expression and chemotherapy resistance. (G) The relationship between *c-Myc* expression and patients' clinical information (n=663). (H) Scheme showing screening for compounds that selectively suppress the growth of c-Myc-KO cells. (I-J) The cell viability of c-Myc-KO and c-Myc-WT KYSE450 cells after treatment with 768 compounds (each at 20μM) (I) or 18 possible compounds (each at 10μM) for 24 hours(J). (K) Cell viability of si control or si *c-Myc* KYSE450 cells (upper) and PLVX or c-Myc-OE KYSE30 cells (lower) after treatment for 24 hours with SGC2085 in different concentration. (L) Kaplan-Meier survival curve showing that expression of *CARM1* uncorrelated with lower disease-free survival in patients with ESCC (P=0.76). (M-N) Growth curves were measured using Incucyte or CCK-8 to analyse KYSE450 or KYSE510 cells stably transfected with control vector (black) or CARM1-knockdown vector (blue and yellow) for 96 h.

The overexpression of *c-Myc*, an important oncogene, contributes to tumorigenesis. *C-Myc* was significantly amplified in various tumors, including esophageal cancer **(Fig. 1A)**. As a transcription factor, *c-Myc* is an intrinsically disordered protein with no stable conformation or suitable binding site for small molecules 26. *c-Myc* is inherently unstable and achieves a stable conformation only when it combines with Max. The c-Myc-Max complex then binds to the E-box sequence on DNA to initiate the transcription of downstream genes 27, 28. Currently, all drugs targeting *c-Myc* have failed in clinical trials, highlighting the urgent need for effective therapeutic strategies.

Using whole-genome sequencing data from 663 cases of ESCC collected in our laboratory, we found that *c-Myc* had a 16.4% amplification rate **(Fig. 1B)**. In addition, based on *c-Myc* amplification, we divided the patients into two groups and observed that patients in the *c-Myc* amplification group had significantly poorer outcomes than those without **(Fig. S1A)**. In contrast, we analyzed tumor and normal tissues and revealed that *c-Myc* expression was significantly higher in tumors than in normal tissues** (Fig. 1C and Fig. S1B)**. Furthermore, we analyzed the expression levels of c-Myc in esophageal tissue from normal mice and esophageal squamous cell carcinoma mice induced by 4NQO using multiplex immunofluorescence technology. We found that the expression levels of c-Myc in the esophagus of ESCC mice were significantly higher than those in normal mice, which is consistent with the results in esophageal cancer patients **(Fig. 1D)**. Moreover, we analyzed the relationship between c-Myc expression and patients' clinical information. We found that *c-Myc* amplification increased the likelihood of metastasis. High *c-Myc* expression was significantly associated with chemotherapy resistance **(Fig. 1E-G)**. Overall, both amplification and high expression of *c-Myc* were associated with poor patient prognosis.

Currently, none of the drugs targeting *c-Myc* have achieved satisfactory clinical results, underscoring the urgent need for improved therapeutic strategies 29. In our subsequent studies, we used CRISPR-Cas9 technology to knock down *c-Myc* in the c-Myc-overexpressing KYSE450 cell line **(Fig. S1C and D)**. To identify drugs that differentially affect c-Myc-wild-type (WT) and c-Myc-knockout (KO) cells, we screened a library of 768 single-target small-molecule inhibitors using a drug concentration of 20 µM and assessed cell viability after 24 h **(Fig. 1H)**. Notably, 11 and 7 drugs were more effective in the c-Myc-WT and c-Myc-KO groups, respectively **(Fig. 1I and Fig. S1E)**. Further screening using siRNA** (Fig. S1F and G)** revealed that the *CARM1* inhibitor SGC2085 had a significant effect in the c-Myc-KO group **(Fig. 1J)**. Subsequently, *c-Myc* was overexpressed in KYSE30 cells with low *c-Myc* expression **(Fig. S1H)**, which showed that the *CARM1* inhibitor, SGC2085, was more effective at lower *c-Myc* expression levels in both KYSE450 and KYSE30 cell lines** (Fig. 1K)**. Additionally, in the KYSE30 cell line, another inhibitor of *CARM1*, EZM2302 30, demonstrated improved efficacy in the low *c-Myc* expression group** (Fig. S1I)**.

*CARM1*, a protein arginine methyltransferase, is an oncogene. Our laboratory data showed that *CARM1* was not associated with survival **(Fig. 1L)**. We constructed *CARM1* knockdown cells in the KYSE450 and KYSE510 cell lines. CCK8 and Incucyte assays were performed, which revealed a significant inhibition of cell growth following *CARM1* knockdown **(Fig. 1M-N)**. These results indicate that *CARM1* inhibitors demonstrate superior efficacy in the low *c-Myc* expression groups.

### Combination of *CARM1* and *c-Myc* inhibitors significantly inhibits ESCC proliferation

**Figure 2:**
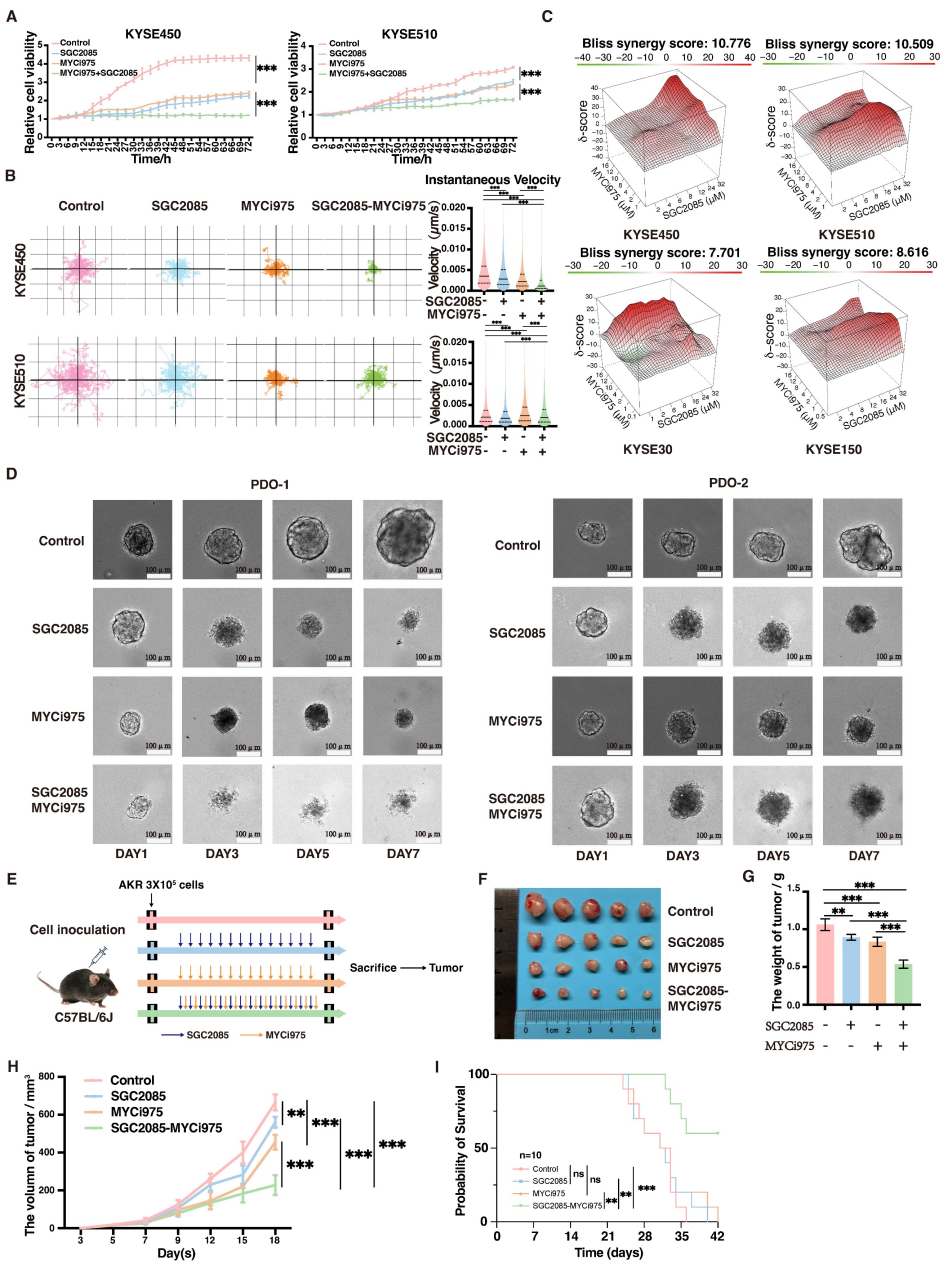
** Combination of *CARM1* and *c-Myc* inhibitors significantly inhibits esophageal squamous carcinoma cell proliferation.** (A) Growth curves were measured using Incucyte live-cell analyses of KYSE450 (left) or KYSE510 (right) cells in control or medicated groups for 72 h. (B) Random motility displacement and track speed of each cell type in the control and medicated groups in KYSE450 (upper) and KYSE510 (lower) cells. The experiments were performed by using Livecyte (Phasefocus, UK) and data were analyzed using two-tailed t-tests. (C) Bliss synergy scores for SGC2085 and MYCi975 in KYSE450, KYSE510, KYSE30, KYSE150 cells. Cells were treated with drugs at the indicated concentrations for 48 h. (D) The combination of SGC2085 and MYCi975 in PDO. (E) The process of animal experiments. (F-H) Macroscopic appearance (F), weights (G), volumes (H) of AKR cell xenografts in control and medicated groups (n=5 per group). Data were analyzed using unpaired t-tests. *P < 0.05, **P < 0.01, and ***P < 0.001. (I) Overall survival of AKR cell xenografts in control and medicated groups (n=10 per group). Data were analyzed using unpaired t-tests. *P < 0.05, **P < 0.01, and ***P < 0.001.

Through screening, we found that SGC2085, a *CARM1* inhibitor, was effective in c-Myc-deficient cells. To address the clinical challenge of targeting *c-Myc* drug deficiency, we attempted drug conjugation. We selected ESCC cell lines KYSE30, KYSE150, KYSE450, KYSE510, and AKR for our experiments. First, we examined the drug sensitivity of these five cell lines to SGC2085, a *CARM1* inhibitor, and MYCi975, a *c-Myc* inhibitor. KYSE30 and KYSE150 cells, which have lower *c-Myc* expression levels, were more sensitive to SGC2085, whereas KYSE450 and KYSE510 cells were not** (Fig. S2A)**. Conversely, KYSE450 and KYSE510 cells, which have higher *c-Myc* expression levels, were more sensitive to MYCi975 **(Fig. S2B)**. Next, we evaluated the combination of SGC2085 and MYCi975 *in vitro* and observed significant inhibition of ESCC proliferation using Incucyte, Livecyte, and plate cloning assays** (Fig. 2A and S2C-E)**. Using psychographic QPI label-free imaging technique, we visualized and measured cell movement, which revealed that the drug combination decreased the track speed and mobility of ESCC cells **(Fig. 2B)**. Additionally, we tested the two inhibitors separately at different concentrations and calculated the co-administration index, which showed a better combined effect of both inhibitors in all four cell lines **(Fig. 2C)**. We also performed drug combination experiments in two organoids, maintaining the concentration of MYCi975 at 10 μM in the medium and SGC2085 at 25 μM, and photographed them on days 1, 3, 5, and 7. The control group showed significant growth and remained healthy, whereas the groups treated with either drug alone exhibited crumpling or deterioration. In contrast, the combination group showed complete necrosis and disintegration of the organoids **(Fig. 2D)**.

For the *in vivo* experiments, AKR cells (3 × 10^5^ cells each) were injected subcutaneously into C57BL/6J mice, and treatment was initiated on day 5 post-injection. SGC2085 (100 mg/kg) was administered orally for 14 consecutive days, and MYCi975 (100 mg/kg) was administered intraperitoneally for 14 consecutive days **(Fig. 2E)**. Although both drugs exhibited anti-tumor effects, tumor volume and weight were significantly lower in the combination group than in the control and individual drug groups **(Fig. 2F-H)**. Furthermore, the combination of SGC2085 and MYCi975 extended mouse survival time than SGC2085 or MYCi975 alone **(Fig. 2I)**. Also, the drug combination had no significant effect on the body weight of mice, with no notable changes in the heart, liver, spleen, lungs, or kidneys, indicating that there is no increase in toxicity **(Fig. S2F and G)**.

In summary, the combination of the *CARM1* inhibitor SGC2085 and the *c-Myc* inhibitor MYCi975 significantly inhibited the proliferation of ESCC cells and did not increase toxicity.

### *CARM1* and *c-Myc* form a transcription complex with p300

To explore the tumor-suppressive roles of SGC2085 and MYCi975, we constructed *c-Myc* knockdown and *CARM1* knockdown cells in the KYSE450 cell line. Knocking down *c-Myc* did not significantly alter CARM1 **(Fig. S3A)**. Similarly, *CARM1* knockdown did not significantly affect c-Myc expression **(Fig. S3B)**. Additionally, when *c-Myc* was overexpressed in the KYSE30 cell line, Western blot analysis confirmed a significant increase in c-Myc protein, whereas CARM1 remained unchanged **(Fig. S3C)**. In addition, consistent results were observed at the RNA level **(Fig. S3D-F)**. Taken together, *c-Myc* and *CARM1* do not directly affect each other but may co-regulate downstream signaling.

**Figure 3:**
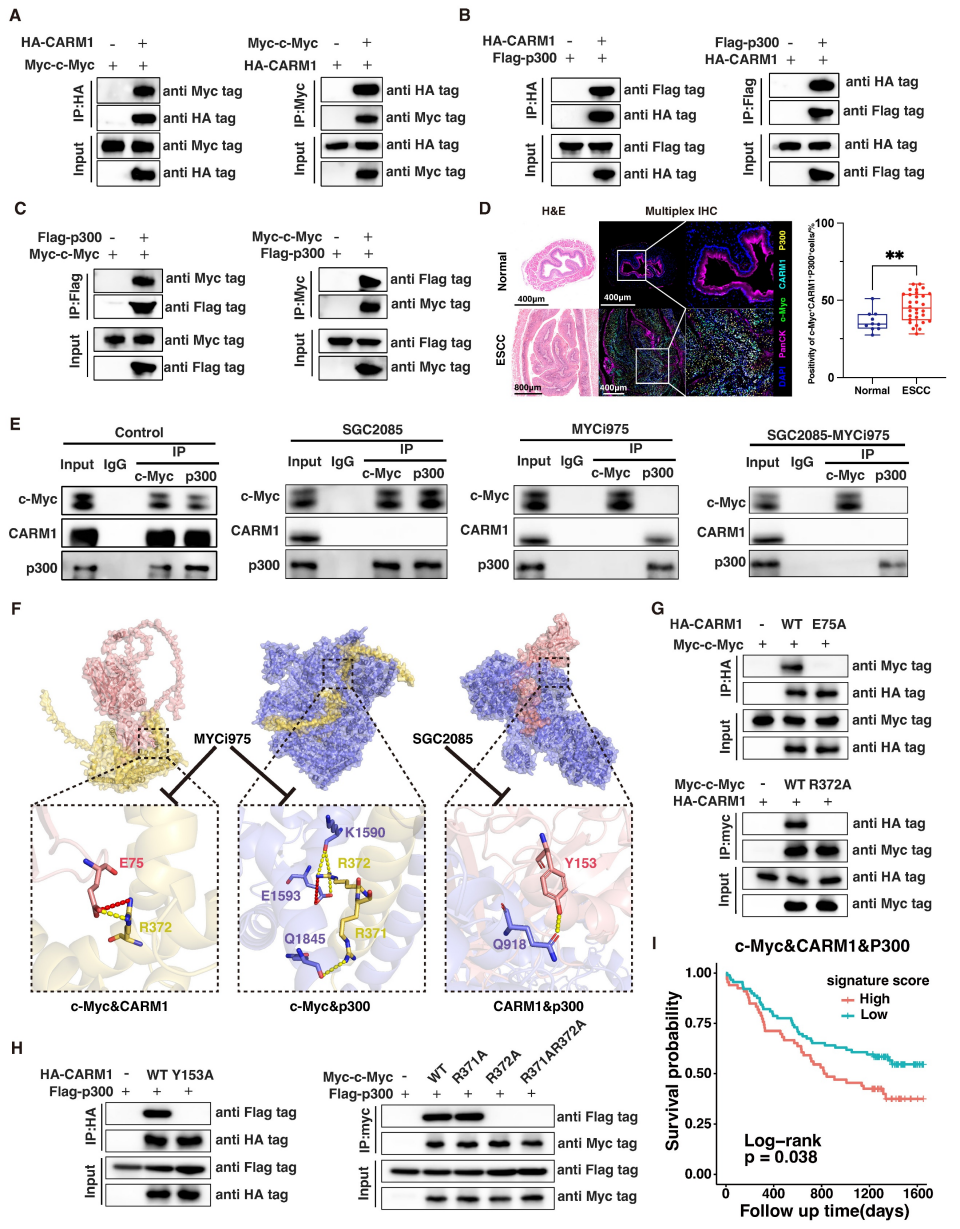
** CARM1 and c-Myc form a transcription complex with p300.** (A-C) Exogenous Co-IP assay of c-Myc and CARM1 (A), p300 and CARM1 (B), c-Myc and p300 (C). Myc-c-Myc, Flag-p300, and HA-CARM1 plasmids were co-transfected into HEK 293 T cells. The immunoprecipitates were analyzed by Western blot using anti-Myc, anti-Flag, and anti-HA antibodies. (D) Representative mIHC image and matched false hematoxylin and eosin images showing the expression of indicated marker genes in esophageal tissue from normal mice and esophageal squamous cell carcinoma mice induced by 4NQO (n=7). (E) Endogenous Co-IP assay of c-Myc, CARM1, and p300 before or after using SGC2085 and MYCi975. The immunoprecipitates were analyzed by Western blot using anti-c-Myc, anti-CARM1, and anti-p300 antibodies. (F) Molecular docking model of c-Myc interacting with CARM1 and p300, CARM1 interacting with p300. (G-H) Exogenous Co-IP assay of c-Myc and CARM1 (G), p300 and CARM1, c-Myc and p300 (H). Myc-c-Myc, Myc-c-Myc-R371A, Myc-c-Myc-R372A, Myc-c-Myc-R371A-R372A, Flag-p300, HA-CARM1, HA-CARM1-E75A and HA-CARM1-Y153A plasmids were co-transfected into HEK 293 T cells. The immunoprecipitates were analyzed by Western blot using anti-Myc, anti-Flag, and anti-HA antibodies. (I) Kaplan-Meier survival curve showing that high expression of *c-Myc*, *CARM1,* and *p300* correlated with lower disease-free survival in patients with ESCC (P=0.038).

In colon cancer cells, c-Myc was amplified and dimerized with Max, and the c-Myc-Max heterodimer recruited p300 and CARM1 to assemble the CPCM complex 31. To validate the interaction between c-Myc and CARM1, we co-transfected Myc-c-Myc and HA-CARM1 into HEK293T cells and performed coimmunoprecipitation (co-IP) using anti-Myc and anti-HA magnetic beads, respectively. The co-IP analysis confirmed that c-Myc can specifically and directly bind to CARM1 **(Fig. 3A)**. Similarly, CARM1 binds to p300, and p300 binds to c-Myc** (Fig. 3B and C)**. We analyzed the co-localization of the three components of the complex change in esophageal tissue from normal mice and 4NQO-induced mice by using multiplex immunofluorescence technology. We can clearly observe that the co-localization in tumors is significantly higher than that in normal tissues. This suggests that we should pay attention to the role of this complex in tumors **(Fig. 3D)**. Further validation of the association between c-Myc, CARM1, and p300 was performed using co-IP assays in KYSE450 cells with anti-c-Myc and anti-p300 antibodies **(Fig. 3E)**. In other words, c-Myc, CARM1, and p300 form a complex that co-regulates downstream signaling.

Concurrent use of SGC2085 and MYCi975 disrupts the interaction between c-Myc, CARM1 and p300, leading to disruption of the transcriptional complex, which inhibited its functional regulation downstream essential for tumor suppression** (Fig. 3E and Fig. S3G)**. MYCi975 targets sites 371-381 on c-Myc, whereas SGC2085 binds to the pocket structure of CARM1. Through molecular virtual docking, we found that c-Myc binds to p300 at position R371 and to both CARM1 and p300 at position R372, which is the target site for MYCi975. Additionally, CARM1 binds to p300 at position Y153, which is the target site for SGC2085. Consequently, this drug combination affects transcriptional complex function, thereby contributing to tumor suppression. We also validated our findings in molecular docking using co-IP **(Fig. 3F-H)**. Our findings indicated that patients with high expression levels of c-Myc, CARM1, and p300 experienced worse survival outcomes **(Fig. 3I)**.

### Combination of *CARM1* and *c-Myc* inhibitors significantly upregulates arachidonic acid levels and inhibits pyrimidine synthesis

**Figure 4:**
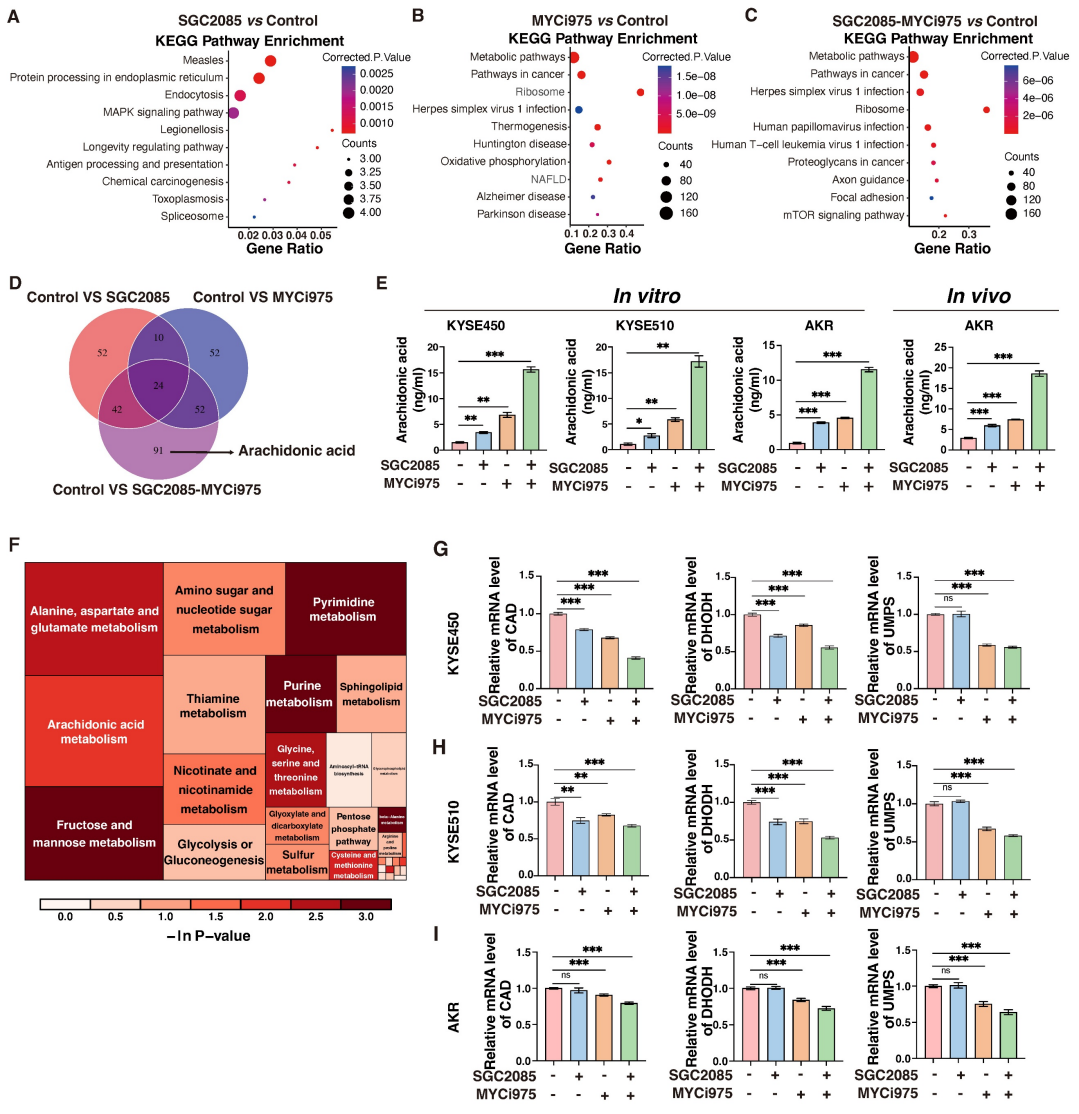
** Combination of *CARM1* and *c-Myc* inhibitors significantly upregulates arachidonic acid levels and inhibits pyrimidine synthesis.** (A-C) KEGG analysis based on the RNA-seq data. (D) Venn diagram showing the changing metabolites between control and medicated groups. (E) ELISA measures the level of AA (Arachidonic Acid) in control and medicated groups in KYSE450, KYSE510 and AKR cells *in vitro* or AKR cells *in vivo*. Data were presented as mean±SD; n=3. Two-tailed t-tests. (F) Heatmap of the changing metabolic pathway. (G-I) RT-qPCR analyses of the *CAD*, *DHODH,* and *UMPS* levels in control and medicated groups in KYSE450 (G), KYSE510 (H) and AKR(I) cells. Data were presented as mean±SD; n=3. Two-tailed t-tests.

Using RNA-sequencing analysis, we found that the pathways significantly enriched in KEGG analysis after drug administration were primarily metabolism-related pathways** (Fig. 4A-C)**. Therefore, we performed additional non-targeted metabolomic testing and found that 209 metabolites were significantly altered in the combination group than in the control group. Among the 91 metabolites that remained unchanged in the individual drug group, arachidonic acid was of particular interest** (Fig. 4D)**. Arachidonic acid is a short-chain unsaturated fatty acid strongly associated with ferroptosis and anti-tumor immunity 32. In the KYSE450 cell line, Enzyme-linked immunosorbent assay (ELISA) demonstrated an increase in arachidonic acid levels in the individual drug group. Interestingly, the combination of *CARM1* and *c-Myc* inhibitors significantly upregulated arachidonic acid levels. Similar results were obtained using ELISA in the KYSE510 and AKR cell lines. What's more, the levels of arachidonic acid significantly increased after the combination of drugs in *in vivo* experiments. **(Fig. 4E)**.

Arachidonic acid is mainly derived from membrane phospholipids catabolized by phospholipase A_2_ (*PLA_2_*), which is regulated by phospholipase A_2_ activating protein (*PLAA*) 33. In the KYSE450 and KYSE510 cell lines, both *PLA_2_* and *PLAA* were significantly upregulated in the drug-associated group using quantitative polymerase chain reaction (qPCR) assay. Arachidonic acid is activated to arachidonoyl coenzyme A (AA-CoA) by acyl coenzyme A synthetase long-chain family protein 4 (*ACSL4*), and the activated lipid molecule is esterified with phosphatidylcholine to produce arachidonic acid-phosphatidylethanolamine (AA-PE) catalyzed by lysophosphatidyltransferase 3 (*LPCAT3*), followed by lipid peroxidation, mediated by the lipoxygenase (*LOX*) family of enzymes 34. In the KYSE450 and KYSE510 cell lines, we examined the profiles of some key enzymes involved in arachidonic acid-metabolizing enzymes, particularly *5-LOX*, which were significantly upregulated following drug treatment **(Fig. S4A-C)**. These findings suggest that the combination of *CARM1* with *c-Myc* inhibitors significantly upregulates arachidonic acid levels.

Metabolic pathway analysis of differential metabolites revealed that pyrimidine metabolism was significantly altered by drug conjugation **(Fig. 4F)**. *De novo* pyrimidine biosynthesis comprises six chemical reactions catalyzed by three enzymes, *CAD*, *DHODH*, and *UMPS*. *CAD*, a multifunctional enzyme that includes carbamoyl phosphate synthetase 2 (CPSII), aspartate transcarbamoylase (ATCase), and dihydroorotate lyase (DHOase), catalyzes the production of dihydroorotate from glutamine. Subsequently, *DHODH* catalyzes the conversion of dihydroorotic acid to orotate and transfers electrons to the electron transport chain to produce ATP. Finally, *UMPS* catalyzes the transfer of the ribonucleic acid phosphate group from ribonucleic acid 5-phosphate-1-pyrophosphate to orotate to form whey protein-5′-monophosphate (orotate-P), which is ultimately decarboxylated to form uridine monophosphate (UMP). *UMPS* catalyzes the conversion of orotate to UMP, which is the predominant form of orotate 35. In the KYSE450 cell line, we found that three key enzymes, *CAD*, *DHODH*, and *UMPS*, were significantly downregulated in the combination group by qPCR assays **(Fig. 4G)**. Similar changes were observed in the ESCC cell line, KYSE510 and AKR** (Fig. 4H and I)**. Therefore, *CARM1*, in combination with *c-Myc* inhibitors, significantly inhibited *de novo* pyrimidine biosynthesis.

In summary, *CARM1*, in combination with *c-Myc* inhibitors, significantly upregulated arachidonic acid levels and inhibited *de novo* pyrimidine biosynthesis.

### Combination of *CARM1* and *c-Myc* inhibitors promotes ferroptosis in ESCC cells

**Figure 5:**
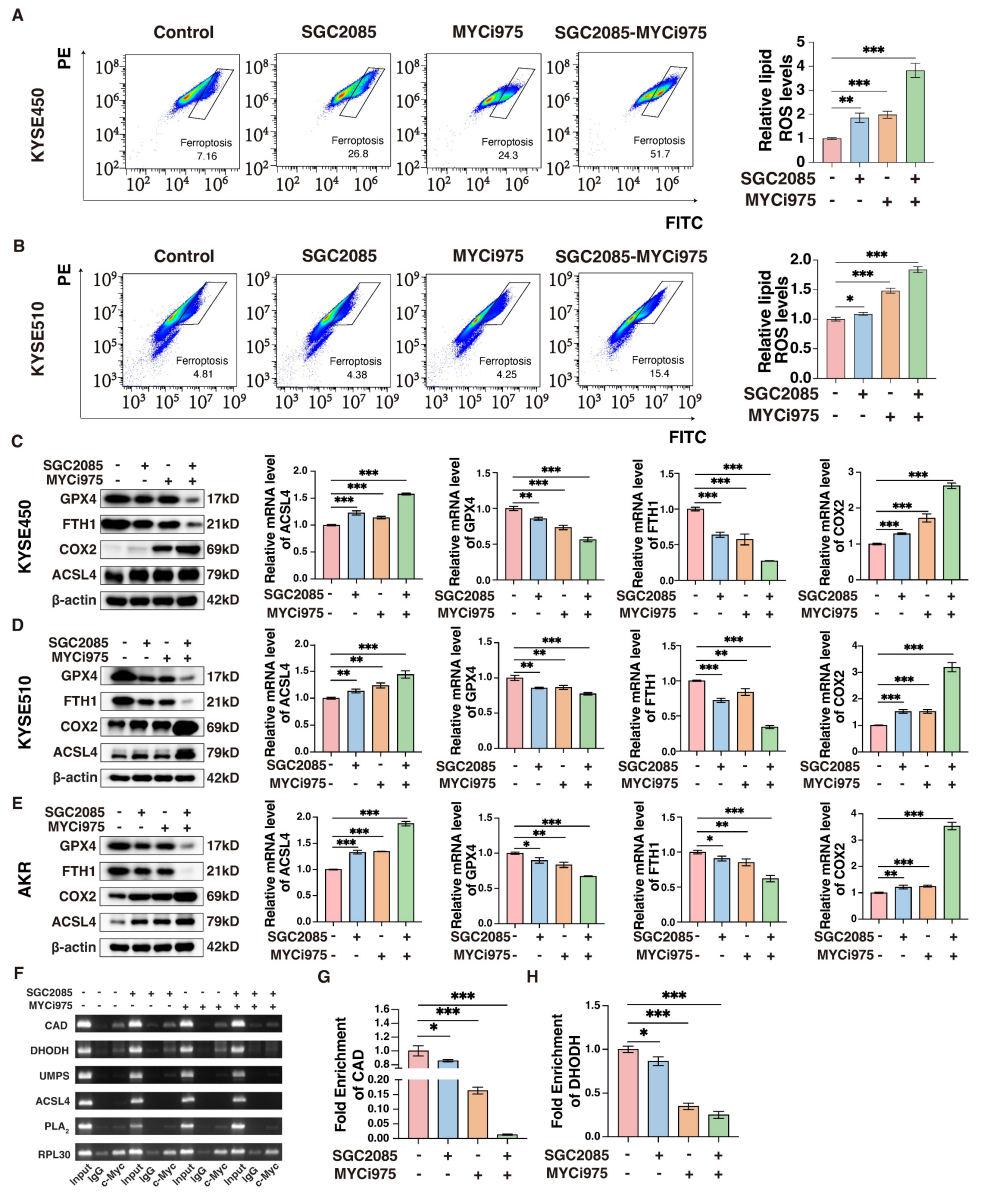
** Combination of *CARM1* and* c-Myc* inhibitors promotes ferroptosis in esophageal squamous carcinoma cells.** (A-B) Lipid ROS levels in control and medicated groups in KYSE450 (upper) and KYSE510 (lower) cells were determined by flow cytometry. Data were presented as mean±SD; n=3. Two-tailed t-tests. (C-E) Western blot or RT-qPCR analyses of the *GPX4*,* ACSL4, FTH1* and *COX2* levels in control and medicated groups in KYSE450 (C), KYSE510 (D) and AKR(E) cells. Data were presented as mean±SD; n=3. Two-tailed t-tests. (F) Agarose gel electrophoresis shows the binding of *c-Myc* to the promoter region of *CAD*, *DHODH*, *UMPS*, *ACSL4,* and *PLA_2_* in KYSE450 cells. (G-H) ChIP-qPCR analyses of the fold enrichment of *CAD* (G) and *DHODH* (H) in control and medicated groups in KYSE450 cells. Data were presented as mean±SD; n=3. Two-tailed t-tests.

Impaired pyrimidine synthesis can lead to ferroptosis 36, which is characterized by lipid peroxidation. In the KYSE450 cell line, flow cytometry analysis revealed that the basal ferroptosis rate was 7.16% after using ferroptosis activators alone without inhibitors, whereas it increased to 51.7% in the combination group. In addition, average fluorescence intensity, which is the gold standard for determining lipid peroxidation, confirmed significantly higher lipid peroxidation levels in the combination group** (Fig. 5A)**. Similar results were observed in the ESCC line, KYSE510** (Fig. 5B)**. In addition, some markers of ferroptosis were significantly altered at the protein and RNA levels; *GPX4* and *FTH1* were significantly downregulated in the combination group, whereas *ACSL4* and *COX2* were upregulated in the combination group **(Fig. 5C-E)**.

To investigate the effects of the drug combination on *de novo* pyrimidine biosynthesis, we hypothesized that the transcription factor *c-Myc* directly binds to certain key metabolic enzymes, specifically *CAD* and *DHODH*. Chromatin immunoprecipitation (ChIP) assay was performed to confirm this direct interaction, which revealed that the binding was significantly weakened following the addition of the inhibitors. In particular, the combination of the drugs further reduced the binding of *c-Myc* to these enzymes **(Fig. 5F)**. The results of ChIP-qPCR confirmed that drug administration significantly affected the binding of *c-Myc* to *CAD* and *DHODH* **(Fig. 5G and H)**. Therefore, we propose that *c-Myc* can directly regulate *CAD* and *DHODH*, and the inhibitors disrupt the transcriptional complex, affecting the binding of *c-Myc* to the corresponding metabolizing enzymes, ultimately affecting *de novo* pyrimidine biosynthesis and, consequently, ferroptosis.

### Combination of *CARM1* and *c-Myc* inhibitors significantly enhances CD8^+^ T cell infiltration within tumors

**Figure 6:**
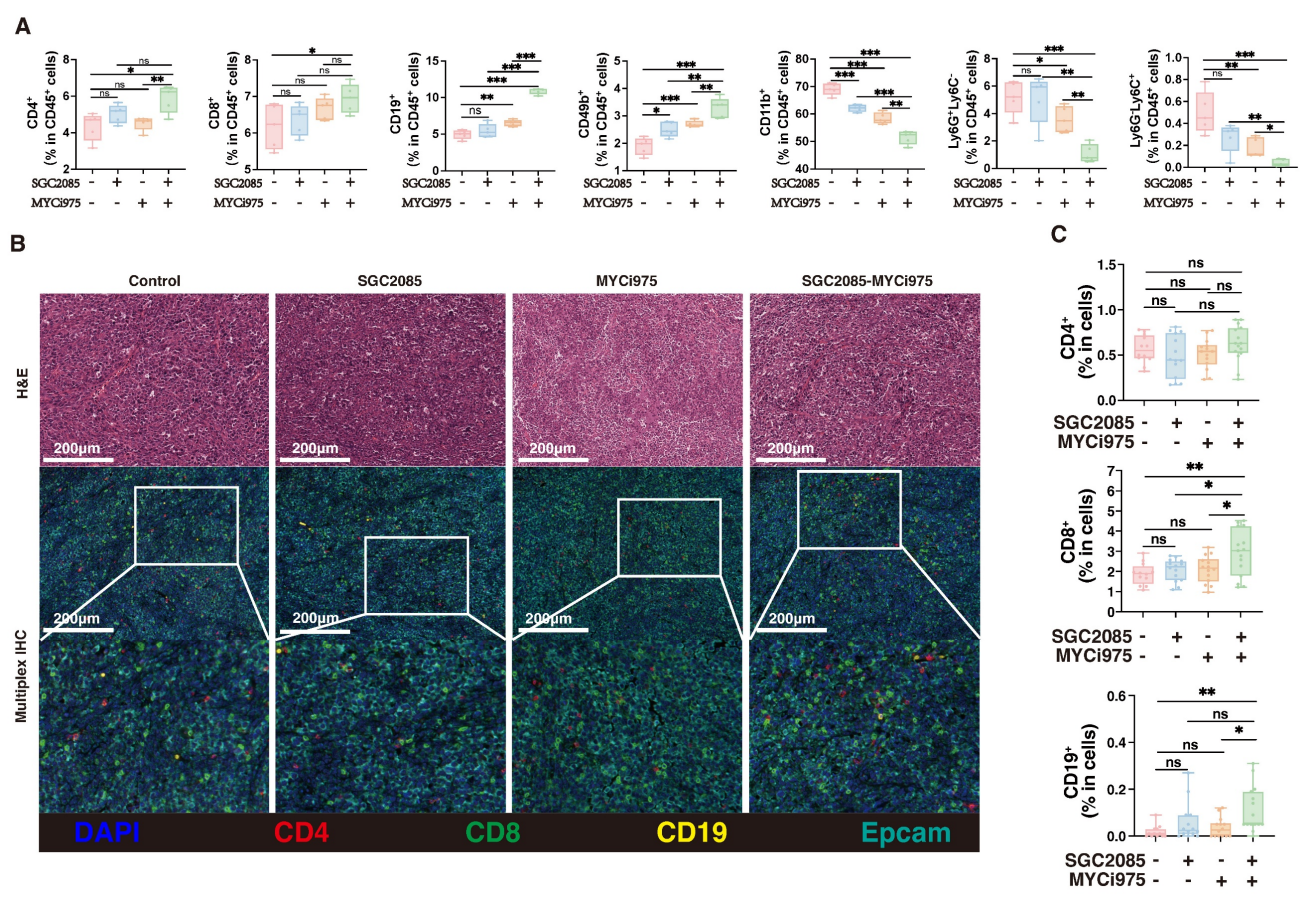

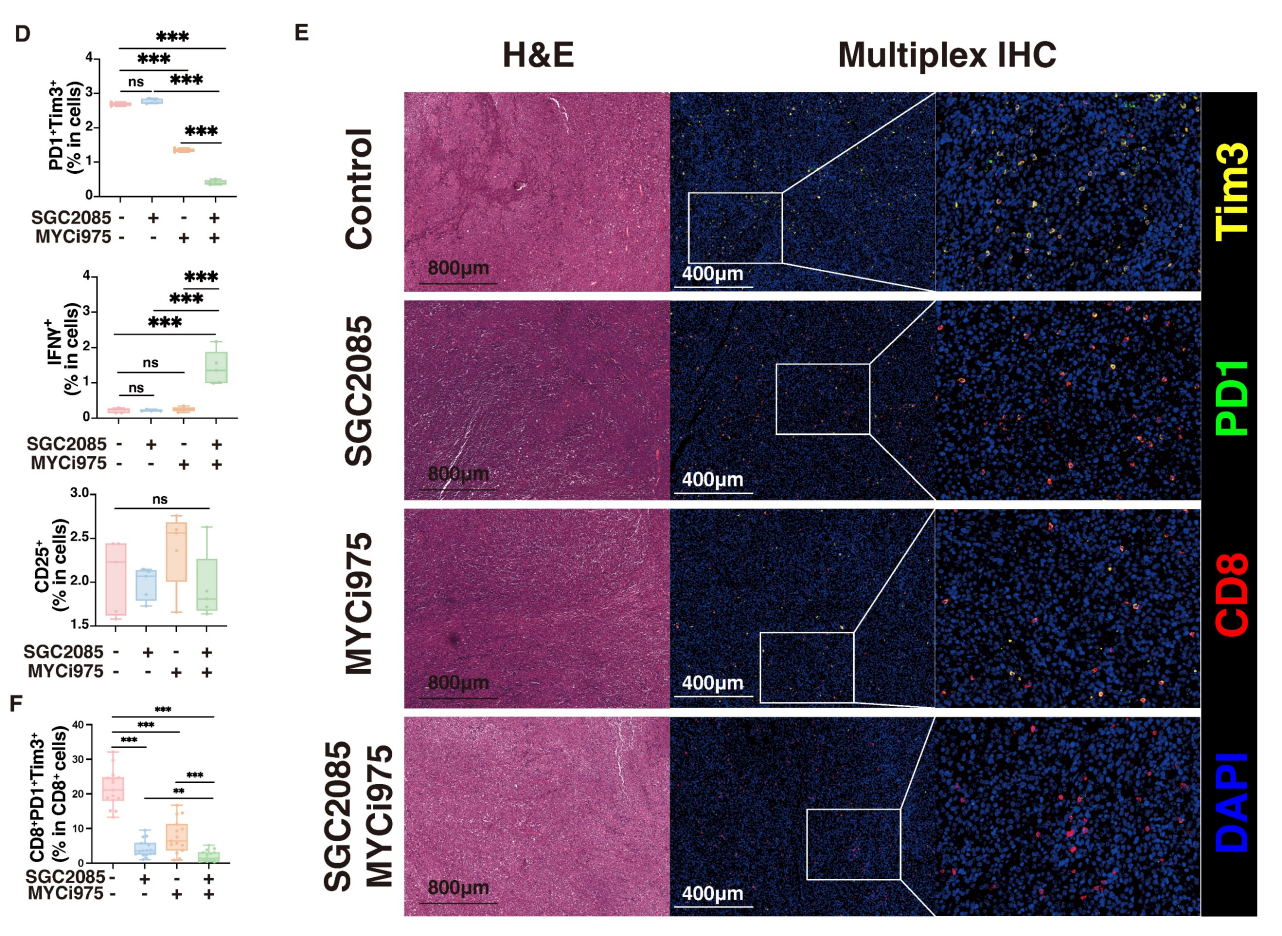
** Combination of *CARM1* and *c-Myc* inhibitors significantly enhances CD8^+^ T cell infiltration within tumors.** (A) Percentage of CD4^+^T, CD8^+^T, CD19^+^B, CD49^+^, CD11b^+^, Ly6G^+^Ly6C^-^ and Ly6G^-^Ly6C^+^ cells in CD45^+^ cells (isolated from the tumor) in control and medicated groups determined by flow cytometry. (B) Representative mIHC image and matched false hematoxylin and eosin images showing the expression of indicated marker genes in tumor tissue both in control and medicated groups. (C) Boxplots indicate the ratio of CD4^+^T, CD8^+^T, and CD19^+^ B cells in tumor tissue both in control and medicated groups. (D) Percentage of PD1^+^Tim3^+^T, IFNγ^+^T, or CD25^+^T cells (isolated from the tumor) in control and medicated groups determined by flow cytometry. (E) Representative mIHC image and matched false hematoxylin and eosin images showing the expression of indicated marker genes in tumor tissue both in control and medicated groups. Boxplots indicate the ratio of CD8^+^PD1^+^Tim3^+^ cells in tumor tissue both in control and medicated groups.

Ferroptosis is closely associated with the tumor immune microenvironment 37. To investigate the impact of our drug combination in the tumor microenvironment that could promote ferroptosis, we performed flow cytometry analysis of the collected mouse subcutaneous tumors and found that CD4^+^ T cells were not significantly altered after drug conjugation, whereas CD8^+^ T cells, B cells and CD49^+^ cells were significantly increased, CD11b^+^ cells, neutrophils and MDSC were significantly decreased** (Fig. 6A)**. Multiple immunofluorescence staining showed that the percentage of CD4^+^ T cells was not significantly changed after drug combination treatment, whereas the percentage of CD8^+^ T cells was significantly increased, contributing to anti-tumor effects. In addition, B cells increased the anti-tumor response **(Fig. 6B and C)**. These findings suggest that the combination of *CARM1* and *c-Myc* inhibitors significantly enhanced the infiltration of CD8^+^ T cells within the tumor, thereby counteracting tumor growth.

To determine which specific subpopulations of CD8^+^ T cells were affected by the drug combination, we analyzed them using flow cytometry. We found that PD1^+^Tim3^+^CD8^+^ T cells were significantly decreased, indicating reduced CD8^+^ T cell exhaustion, whereas IFNγ^+^CD8^+^ T cells were significantly increased, indicating enhanced cytotoxic activity **(Fig. 6D and Fig. S5)**. We also analyzed the exhaustion of CD8^+^ T cells after different therapy using multiplex immunofluorescence technology. We can clearly observe a significant reduction in the level of CD8^+^PD1^+^Tim3^+^ cells in the combination therapy group, which is consistent with the flow assay **(Fig. 6E)**. Therefore, the drug combination significantly decreased CD8^+^ T cell exhaustion and enhanced cytotoxic activity.

**Figure 7:**
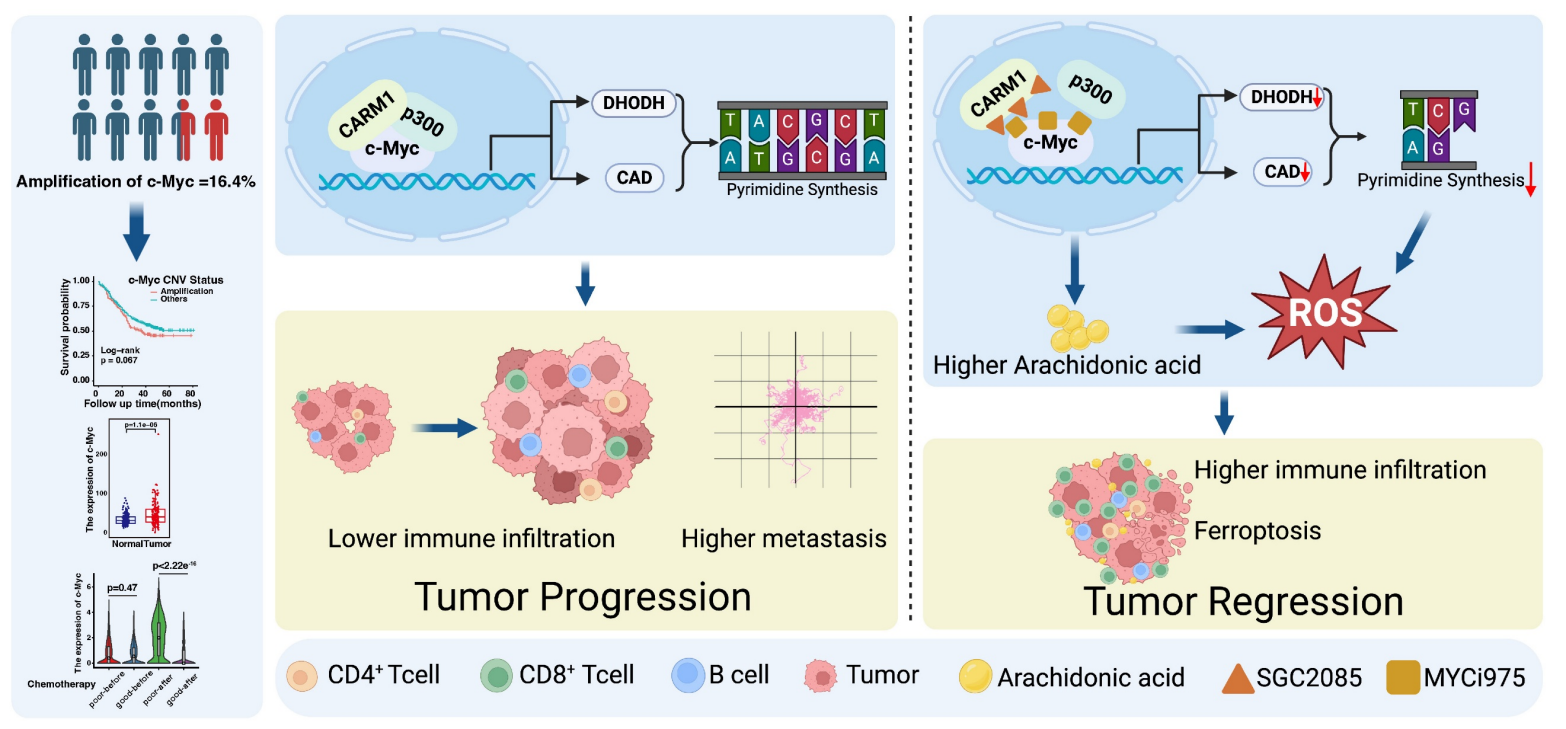
** Targeting c-Myc-p300-CARM1 complex induces ferroptosis and reduces CD8^+^ T cell exhaustion in esophageal squamous cell carcinoma.** Summary schematic of this article. Schematic was created with BioRender (www.biorender.com).

In summary, these findings highlight the therapeutic potential of combining MYCi975 (a *c-Myc* inhibitor) with SGC2085 (a *CARM1* inhibitor) for ESCC. The drugs disrupt the transcriptional complex formed by c-Myc, CARM1, and p300 and regulate the downstream arachidonic acid metabolism and *de novo* pyrimidine biosynthesis. Moreover, it promotes ferroptosis and infiltration of CD8^+^ T cells within the tumor and reduces CD8^+^ T cell exhaustion. In conclusion, we developed a strategy to combine drugs to improve the survival of patients with ESCC **(Fig. 7)**.

## Discussion

Therapeutic strategies targeting *MYC* include direct treatment with *MYC* inhibitors for patients with MYC-amplified tumors, utilization of drugs targeting specific gene products for patients with synthetic lethal targets of *MYC*, MYC-directed immunotherapy for tumors with suppressed immune cells, and metabolic pathway-targeting drugs for tumors with MYC-induced metabolic dysregulation 7-11. However, current clinical agents targeting *c-Myc* have demonstrated poor outcomes, highlighting the urgent need for developing effective therapeutic strategies.

*MYC* regulates various metabolic pathways in tumor cells and substantially influences immune function through its effects on metabolism. *MYC* can affect the immune microenvironment through direct or indirect regulation of metabolism 38. MYC-driven tumors disrupt glucose, glutamine, and lipid metabolism, which affects the host metabolic balance and indirectly affects the immune system. During tumor initiation in mouse models, MYC-driven tumor cells release metabolites such as lactate and glutamate, which affect immune function within the microenvironment. As tumors grow, these effects can lead to systemic impairment of the anti-tumor immune response. The drug combination used in our study significantly affected *de novo* pyrimidine biosynthesis and arachidonic acid metabolism. *DHODH* and *CAD*, the key metabolic enzymes involved in pyrimidine synthesis, were significantly downregulated by the drug combination, affecting *de novo* pyrimidine biosynthesis. In addition, arachidonic acid levels were significantly increased, which enhanced lipid peroxidation, thereby promoting ferroptosis.

Ferroptosis is a novel immunogenic mode of cell death in which the immune microenvironment is significantly altered 37. On one hand, ferroptosis can positively enhance antitumor immunity. Activated CD8^+^ T cells could secrete IFNγ, downregulating System X_c_⁻ expression in tumor cells and enhancing ferroptosis 39. Inhibiting GPX4 in regulatory T cells (Tregs) induces their ferroptosis, strengthens T helper 17 cell responses, and weakens immunosuppression 40. On the other hand, ferroptosis may also exert an inverse inhibitory effect on immunity. Prostaglandin E_2_ (PGE_2_) released during ferroptosis in tumor cells inhibits the recruitment of dendritic cells (cDC1s) and natural killer (NK) cells. Oxidized lipids released after ferroptosis in tumor-associated neutrophils (PMN-MDSCs) suppress T cell function 41. These findings provide a balanced approach for combining ferroptosis inducers with immunotherapy. Checkpoint blockade therapy is emerging as a revolutionary therapy that influences the choice of strategies for tumor treatment 42. *MYC* enables tumors to evade the immune response through several mechanisms. *MYC* regulates the expression and production of various immune ligands or receptors and immune effector molecules, such as *PD-L1*, *CD47*, and *MHC class I* 43, 44. Moreover, *MYC* promotes the expression of several cytokines, such as *CCL2*, *IL-23*, and *CCL9*, which regulate the conversion of anti-tumor M1 macrophages to pro-tumor M2 macrophages and prevent the activation and recruitment of B cells, NK cells, and CD4^+^ and CD8^+^ T cells. *CCL9* activates mast cells, which, in turn, induce angiogenesis. Upon *MYC* inactivation, downregulation of *PD-L1* and *CD47* leads to the rapid recruitment and activation of CD8^+^ T cells and NK cells. Furthermore, *MYC* inactivation increases *NKG2DL* levels in cancer cells, leading to NK cell recruitment. The production of these cytokines decreased after *MYC* inactivation. In contrast, increased expression of type I interferon and *CCL5* upon *MYC* inactivation leads to the recruitment and activation of NK cells, B cells and CD8^+^ T cells 45. Therefore, *MYC* controls the immune status of tumors by creating an immunosuppressive cold tumor microenvironment upon activation, which reverts to an immunosensitive hot environment upon inactivation.

Our findings highlighted that the combination of MYCi975 and SGC2085 altered the tumor microenvironment. Using flow cytometry and multiplex immunofluorescence, we found that CD8^+^ T cells and B cells were significantly upregulated in the tumor microenvironment. Notably, CD8^+^ T cell exhaustion was significantly reduced with an increase in the number of cells with cytotoxic functions, indicating activation of the tumor immune microenvironment. Currently, most studies on immune checkpoint inhibitors have focused on checkpoint proteins and T cells infiltration in the tumor microenvironment. This study showed that the drug combination also induced increased T-cell infiltration in the tumor microenvironment. The result suggests a promising treatment strategy that the combination of *c-Myc* and *CARM1* inhibitors could activate immune infiltration in the tumor microenvironment, providing a viable option for chemotherapy-resistant patients. In conclusion, these findings offer valuable insights for future mechanistic research and therapeutic interventions.

## Materials and Methods

### Drug library screen

c-Myc-WT and c-Myc-KO KYSE450 cancer cells were used for screening assays. Cells were seeded in 96-well plates, incubated for 18 h, and then treated with the drug at a concentration of 20 μM (Anti-tumor Inhibitor Kit, including 768 compounds Supplementary Information, Table S1). After 24 h, cell viability was assessed at 450 nm. Candidate compounds were considered if the viability of c-Myc-KO cells/c-Myc-WT cells was < 0.65 or > 1.5 46.

### Cell culture

The human ESCC cell lines were sponsored by Dr. Yutaka Shimada (Kyoto University, Japan). The mouse AKR cells were kindly provided by Dr. Anil Rustgi (Columbia University) 47-49. The HEK293T cell lines were purchased from the American Type Culture Collection (ATCC, Manassas, VA, USA). ESCC cell lines and HEK293T cells were cultured in RPMI-1640 medium and Dulbecco's modified Eagle's medium (DMEM), respectively, both supplemented with 10% fetal bovine serum (FBS) at 37 °C with 5% CO2. All cells were routinely subjected to short tandem repeat analysis and were regularly tested for mycoplasma contamination.

### Organoid culture and drug treatments

Tissues collected from 4-nitroquinoline 1-oxide (4NQO)-induced mice were used to establish patient-derived organoid models. Fresh specimens were immediately rinsed in phosphate-buffered saline (PBS) three times, then dissociated using collagenase/hyaluronidase (STEMCELL Technologies #07912, 1:10; Vancouver, BC, Canada) for 60 min at 37 °C. Subsequently, two-thirds volume of modified Hank's balanced salt solution (STEMCELL Technologies #37150) with 5% FBS was added, followed by centrifugation at 400 × g to collect the cells. The cells were resuspended in organoid culture medium and plated in 24-well plates at a density of 16,000 cells/well. Culture medium was used as previously described 50. Organoids were cultured in organoid culture medium. Subsequently, 25 μM SGC2085 (MCE; HY-100565) and/or 10 μM MYCi975 (Selleck; S8906) were added on alternate days at the indicated concentrations.

### Animal experiments

All animal experiment protocols were approved by the Animal Care and Use Committee of the Chinese Academy of Medical Sciences Cancer Hospital. For subcutaneous xenografting, 3 × 10^5^ AKR cells were implanted into 6-week-old male C57 mice (Vital River Laboratory Animal Technology, Beijing, China). The mice were maintained under normal conditions and were fed standard diet. After 5 days, the mice were randomly assigned to one of the four groups. The tumor length and width were measured using a caliper, and the volume was calculated using the formula 0.5 × length × width^2^. Drug treatment method: Five days after inoculation, the mice received SGC2085 (100 mg/kg), MYCi975 (100 mg/kg), or both (SGC2085 50 mg/kg + MYCi975 50 mg/kg) daily for 14 days. 4NQO-induced mice models were performed as previously described 51.

### Plasmids, lentiviruses, and transduction

Full-length human c-Myc was cloned into the pcDNA3-Myc vector. Full-length human CARM1 was cloned into the pcDNA3-HA vector. Full-length human P300 was cloned into the pcDNA3-Flag vector. The c-Myc and CARM1 shRNA sequences were cloned into the pSIH-H1 vector.

Lentiviruses were produced using HEK293T cells with a second-generation packing system: pMD2.G (#12 259, Addgene) and psPAX2 (#12 260, Addgene). For lentivirus packaging, 1.5 μg pMD2.G, 4.5 μg psPAX2, along with 6 μg pSIH-H1-puro/pSIH-H1-puro-shRNA was co-transfected into HEK293T cells with Liposomal Transfection Reagent (40802, Yeasen, Shanghai, China). After 48 h, the supernatant was harvested, centrifuged at 1000 × g for 10 min at 4 °C, and then filtered using a 0.45-μm syringe filter. For viral transductions, 2.5 × 10^5^ cells/well were seeded in 6-well culture plates and infected with viruses plus polybrene (8 μg/mL) for 48 h.

To obtain stable cell lines, the infected cells were treated with puromycin (1 μg/mL) for 1-2 weeks. The sequences of the siRNAs, shRNAs, and sgRNAs are listed in Supplementary Table S2.

### Antibodies and reagents

Antibodies against the following proteins were used for western blotting: β-actin (1:1000, Cell Signaling Technology; 4970), c-Myc (1:1000, Cell Signaling Technology; 9402), CARM1 (1:1000, Abcam; ab243638), P300 (1:1000, Cell Signaling Technology; 54062), Flag-tag (1:1000, Cell Signaling Technology; 14793), Myc-tag (1:1000, Cell Signaling Technology; 2272), HA-tag (1:1000, Abcam; ab236632), GPX4 (1:1000, Cell Signaling Technology; 52455), ACSL4 (1:1000, Abcam; ab155282), FTH1 (1:1000, Abcam; ab75973), and COX2 (1:1000, Abcam; ab179800).

SGC2085 (MCE; HY-100565), EZM2302 (MCE; HY-111109), MYCi975 (Selleck; S8906), and Erastin (MCE; HY-15763) were used.

### Immunoprecipitation and western blot analysis

Total cell lysates were prepared using radioimmunoprecipitation lysis buffer with freshly added protease inhibitor cocktail (0 469 315 9001, Roche, Basel, Switzerland) and phosphatase inhibitor cocktail (0 490 684 5001, Roche, Basel, Switzerland) for 20 min on ice. For co-IP assays, IP lysis buffer was used. Protein concentrations were quantified using a BCA assay kit (Thermo Scientific). For immunoprecipitation, equal amounts of protein lysate were incubated overnight at 4 °C with anti-HA magnetic beads (MCE; HY-K0201), anti-c-Myc magnetic beads (MCE; HY-K0206), or anti-Flag magnetic beads (M8823, Sigma-Aldrich) for exogenous protein co-IP, and protein A/G magnetic beads (HY-K0202, MCE), anti c-Myc antibody or anti-p300 antibody for endogenous protein co-IP. The beads were washed three times with cell lysis buffer and eluted with 2× loading buffer. Protein extracts were resolved using 6% or 10% sodium dodecyl sulfate-polyacrylamide gel electrophoresis, transferred onto polyvinylidene fluoride membranes (Merck Millipore, MA, USA), and visualized by chemiluminescence.

### RNA isolation and quantitative real-time PCR (qRT-PCR)

Total RNA was extracted from cultured cells using TRIzol reagent (Thermo Scientific, MA, USA). RNA was subsequently reverse-transcribed into complementary DNA using a Quantscript RT Kit (KR103, Tiangen, Beijing, China) according to the manufacturer's instructions. qRT-PCR was performed using PowerUpTM SYBRTM Green Master Mix (A25742, Applied Biosystems, CA, USA), and analysis was performed using a StepOnePlus Real-Time PCR system (Applied Biosystems, CA, USA). The relative expression levels of the target genes were standardized to those of the housekeeping gene, GAPDH. The qRT-PCR primers are listed in Supplementary Table S2.

### Half maximal inhibitory concentration (IC50) and Bliss index analysis

Esophageal cancer cell lines were plated in the same medium for the drug combination experiments. SGC2085 cells were combined with MYCi975 cells in 8 × 8 dose-response matrices in 96-well plates. Esophageal cancer cell lines (KYSE150 and KYSE510) were seeded at 10,000 cells per well, whereas esophageal cancer cell lines (KYSE30 and KYSE450) were seeded at 12,000 cells per well. Relative cell viability was determined using the CCK-8 kit (TargetMol, KA288282) after 48 h of drug treatment, and the absorbance was measured at 450 nm.

For the Bliss matrix synergy experiments, the drug concentrations were selected to ensure similar effects on cellular viability after 48 h of treatment. The goal was to select a range of concentrations with effects ranging from 30 to 100% viability. Concentrations with less than 30% effect were prioritized, because those with stronger effects are less likely to demonstrate synergy due to the dominant effect of one drug.

Drug combination data were analyzed using the SynergyFinder package with the Bliss independence model 52, which converts percent viability values to fraction-affected (FA). The Bliss excess score represents the difference between the expected growth inhibition and the observed inhibition, with score > 0 indicating synergy, close to zero indicating additivity, and < 0 indicating antagonism.

### Live-cell microscopy and quantification and CCK-8 (cell proliferation and cell viability) assays

A total of 2000-4000 cells were seeded in 96-well plates depending on the growth rate and experimental design. Approximately after 16 h, 25 μM SGC2085 (MCE; HY-100565), and/or 10 μM MYCi975 (Selleck; S8906) were added at the indicated concentrations. The cells were imaged every 3 h using an Incucyte S3 microscope (Essen Bioscience) or a Livecyte 419 Phase Focus microscope (Essen Bioscience). Data analysis was performed as previously described 53. CCK-8 assay was performed as previously described 54.

### Colony formation assay

Cells were seeded in 6-well plates at 1500 cells per well and incubated at 37 °C for 2 weeks, and the medium was changed every 3-4 days. The cells were fixed and stained with crystal violet after 2 weeks, and the number of clones was counted.

### RNA-seq and gene expression signature analysis

RNA-seq and gene expression signature analysis were performed as previously described 55.

### ELISA for determining arachidonic acid concentration

Arachidonic acid concentration was determined using an ELISA Kit according to the manufacturer's instructions (Elabscience Biotechnology; E-EL-0051c).

### BODIPY-C11 staining

For BODIPY-C11 staining, the cells were suspended in 1 mL of PBS containing 3 mM BODIPYTM 581/591 C11. The suspension was then incubated for 30 min at 37 °C in a tissue culture incubator. After incubation, the cells were washed and resuspended in 200 μL of PBS. Flow cytometry and other analyses were performed as described previously 56.

### ChIP qPCR

The ChIP assays were performed using the SimpleChIP Enzymatic Chromatin IP Kit according to the manufacturer's instructions (Cell Signaling Technology #9003; Beverly, MA, USA). Briefly, KYSE450 cells were formaldehyde-crosslinked, and the DNA was sheared with the enzyme. The cross-linked whole-cell extract was used for immunoprecipitation with a c-Myc antibody (Cell Signaling Technology; 9402) or control IgG. The samples were incubated overnight with antibodies and rinsed with wash buffer. Quantitative real-time PCR analysis was performed on ChIP-purified DNA using the SYBR Green PCR Master Mix (Applied Biosystems #4309155; Carlsbad, CA, USA), following the manufacturer's protocol. All samples were normalized to IgG as a control.

### Flow cytometry

The mice were dissected, and the tumor tissues were removed. Tumor tissues were digested with enzymes into single-cell suspensions and neutralized with a medium containing 10% FBS. Erythrocytes were lysed with erythrocyte lysing solution, and tumor cells were washed with PBS. The tumor cells were then stained for 30 min. Antibodies used are listed in Supplementary Table S3.

### Multiplex immunofluorescence assay

Multiplex immunofluorescence staining of formalin-fixed and paraffin-embedded tumor sections (4-μm thickness) was performed using a seven-color multi-labeling kit (PANOVUE, TSA-Rab-247259). The sections were deparaffinized in xylene and rehydrated in a series of graded alcohol solutions, including 100%, 95%, 90%, 80%, and 70%. Heat-induced epitope retrieval was performed using slides immersed in boiling sodium citrate buffer for 5 min in a microwave oven. Each primary antibody was incubated for 1 h at 37 °C, followed by the secondary antibody application for 15 min and incubation of the tertiary TSA-amplification reagent for 15 min. Different fluorescence signals from Opal520, Opal570, Opal620, and Opal650 were generated, corresponding to CD8 (Abcam, ab217344), CD4 (Abcam, ab288724), Epcam (Abcam, ab71916), and CD19 (Cell Signaling Technology, 90176), PD1 (Cell Signaling Technology, 84651), Tim3 (Abcam, ab241322), panCK (Abcam, ab7753), c-Myc (Abcam, ab32072), CARM1 (Abcam, ab243638), p300 (Abcam, ab275379). The slides were counterstained with DAPI for 10 min for nuclei visualization and mounted in mounting medium. Multispectral images were acquired using the Vectra Polaris Automated Quantitative Pathology Imaging System (PerkinElmer), with inForm software (AKOYA) used for unmixing signals, removing autofluorescence, and quantifying cells of interest. The percentage of positive cells was scored as > 75%.

### Statistical analysis

All statistical analyses and graph generation were performed using GraphPad Prism version 9.0.1 software (San Diego, CA, USA). Statistical significance was calculated using an unpaired Student's t-test. Correlation analyses were performed using Pearson's correlation coefficients. Each experiment was performed at least three times, and quantitative data are presented as the mean ± standard deviation.

## Supplementary Material

Supplementary figures and tables.
