# Supplementary material for: Targeting c-Myc-p300-CARM1 complex induces ferroptosis and reduces CD8+ T cell exhaustion in esophageal squamous cell carcinoma
Source: Int J Biol Sci. 2026 Jan 1;22(3):1266–82. doi: 10.7150/ijbs.114575 (PMC12837744; doi:10.7150/ijbs.114575)
Supplement: Supplementary file 1 — Supplementary figures and tables. [file ijbsv22p1266s1.pdf]

## Supplementary material

**Table S1.** List of Anti-tumor Inhibitor Kit used in this study.

| Catalog Number | Product Name | Target |
|---|---|---|
| HY-100006A | MRT68921 (dihydrochloride) | ULK |
| HY-100011 | SR-3029 | Casein Kinase |
| HY-100012 | CBR-5884 | PHGDH |
| HY-100017 | BAY-876 | GLUT |
| HY-100018 | BAY1125976 | Akt |
| HY-100019 | Rogaratinib | FGFR |
| HY-100020 | BAY-1436032 | Isocitrate Dehydrogenase (IDH) |
| HY-100023 | LY3177833 | CDK |
| HY-100026 | PQR620 | mTOR |
| HY-100110 | KNK437 | HSP |
| HY-100116A | Mitoquinone (mesylate) | Reactive Oxygen Species |
| HY-100155 | 4-IBP | Sigma Receptor |
| HY-10019 | Varenicline | nAChR |
| HY-100207 | CP21R7 | GSK-3 |
| HY-100213 | EAI045 | EGFR |
| HY-100220 | GSK6853 | Epigenetic Reader Domain |
| HY-100222 | CZ415 | mTOR |
| HY-100233 | IQ-1S (free acid) | JNK |
| HY-100235 | GSK591 | Histone Methyltransferase |
| HY-100343 | GNE-495 | MAP4K |
| HY-100350 | CA-074 methyl ester | Cathepsin |
| HY-100351 | BI-7273 | Epigenetic Reader Domain |
| HY-100352 | BI-9564 | Epigenetic Reader Domain |
| HY-100360 | MS049 | Histone Methyltransferase |
| HY-100368A | MELK-8a (hydrochloride) | MELK |
| HY-100370 | MRK-016 | GABA Receptor |
| HY-100390 | (S)-ML753286 | BCRP |
| HY-100394 | NVP-BAW2881 | VEGFR |
| HY-100403 | Ro 67-7476 | mGluR |
| HY-100408 | GNF-6231 | Porcupine |
| HY-10042 | Odanacatib | Cathepsin |
| HY-100421 | CPI-455 | Histone Demethylase |

| | | |
|---|---|---|
| HY-100422 | KDM5-IN-1 | Histone Demethylase |
| HY-100445A | αvβ1 integrin-IN-1 (TFA) | Integrin |
| HY-100447 | TM5275 (sodium) | PAI-1 |
| HY-100483 | A-804598 | P2X Receptor |
| HY-100488 | Bay 59-3074 | Cannabinoid Receptor |
| HY-100493 | BP-1-102 | STAT |
| HY-100499 | Tyrphostin AG 528 | EGFR |
| HY-100506 | GLPG0187 | Integrin |
| HY-100524 | LRE1 | Adenylate Cyclase |
| HY-100526 | XMU-MP-1 | Hippo (MST) |
| HY-100529 | PD150606 | Proteasome |
| HY-100563 | Cyclo(RGDyK) (trifluoroacetate) | Integrin |
| HY-100565 | SGC2085 | Histone Methyltransferase |
| HY-100573 | Necrosulfonamide | Mixed Lineage Kinase |
| HY-100591 | SirReal2 | Sirtuin |
| HY-100611 | CaCCinh-A01 | Chloride Channel |
| HY-100614 | AS1517499 | STAT |
| HY-10064 | Ticagrelor | P2Y Receptor |
| HY-100653A | AZD5153 (6-Hydroxy-2-naphthoic acid) | Epigenetic Reader Domain |
| HY-100659 | Dihydrodiol-Ibrutinib | Btk |
| HY-100688 | ML390 | Dihydroorotate Dehydrogenase |
| HY-100693 | SYP-5 | HIF/HIF Prolyl-Hydroxylase |
| HY-100716 | Eganelisib | PI3K |
| HY-100742 | GNE-140 (racemate) | Lactate Dehydrogenase |
| HY-100742A | (R)-GNE-140 | Lactate Dehydrogenase |
| HY-100742B | (S)-GNE-140 | Lactate Dehydrogenase |
| HY-100747 | PSB-12379 | CD73 |
| HY-100768 | HTHQ | Reactive Oxygen Species |
| HY-100790 | Ciliobrevin A | Hedgehog |
| HY-100818 | Futibatinib | FGFR |
| HY-10082 | 3-AP | DNA/RNA Synthesis |
| HY-100832 | UNC3866 | Histone Methyltransferase |
| HY-10087 | Navitoclax | Bcl-2 Family |
| HY-100877 | GCN2-IN-1 | Eukaryotic Initiation Factor (eIF) |
| HY-100888 | Simurosertib | CDK |
| HY-100962 | (E)-AG 99 | EGFR |
| HY-101025 | Nrf2-IN-1 | Keap1-Nrf2 |

| | | |
|---|---|---|
| HY-101028 | ZINC13466751 | HIF/HIF Prolyl-Hydroxylase |
| HY-101038A | ZK756326 (dihydrochloride) | CCR |
| HY-101053 | Src Inhibitor 1 | Src |
| HY-101068 | TOFA | Acetyl-CoA Carboxylase |
| HY-10110 | IC-87114 | PI3K |
| HY-10111 | TG100-115 | PI3K |
| HY-101117 | EED226 | Histone Methyltransferase |
| HY-101126 | TP-3654 | Pim |
| HY-10114 | TGX-221 | PI3K |
| HY-101177 | Naltrindole (hydrochloride) | Opioid Receptor |
| HY-101249 | L-732138 | Neurokinin Receptor |
| HY-101267 | CHMFL-BMX-078 | BMX Kinase |
| HY-101272 | GDC-0326 | PI3K |
| HY-101278 | Thiomyristoyl | Sirtuin |
| HY-101280 | LB-60-OF61 | NAMPT |
| HY-101284 | DMU2105 | Cytochrome P450 |
| HY-101285 | DMU2139 | Cytochrome P450 |
| HY-101371 | Hesperin | Keap1-Nrf2 |
| HY-101512 | A-395 | Histone Methyltransferase |
| HY-101513 | eIF4A3-IN-1 | Eukaryotic Initiation Factor (eIF) |
| HY-101524 | TC13172 | Mixed Lineage Kinase |
| HY-101533 | AZD-5991 | Bcl-2 Family |
| HY-101550 | HI-TOPK-032 | TOPK |
| HY-101563 | GSK3326595 | Histone Methyltransferase |
| HY-101566A | Elimusertib (hydrochloride) | ATM/ATR |
| HY-101568 | Roblitinib | FGFR |
| HY-101572 | MHP | SphK |
| HY-101611 | MSC2530818 | CDK |
| HY-10172 | IMD-0354 | IKK |
| HY-101769 | UAMC00039 (dihydrochloride) | Dipeptidyl Peptidase |
| HY-101770 | BTB-1 | Microtubule/Tubulin |
| HY-101774 | CSF1R-IN-1 | c-Fms |
| HY-101778 | ML311 | Bcl-2 Family |
| HY-101790A | ZM223 (hydrochloride) | NEDD8-activating Enzyme |
| HY-101799 | NCGC00244536 | Histone Demethylase |
| HY-10180 | MLN8054 | Aurora Kinase |
| HY-101844 | ML-18 | Bombesin Receptor |

| | | |
|---|---|---|
| HY-101845 | FITM | mGluR |
| HY-101846 | AX-15836 | ERK |
| HY-101853 | STAT5-IN-1 | STAT |
| HY-101864 | Blarcamesine (hydrochloride) | Sigma Receptor |
| HY-101870B | Uzansertib (phosphate) | Pim |
| HY-101928 | KDM4D-IN-1 | Histone Demethylase |
| HY-101931 | hVEGF-IN-1 | VEGFR |
| HY-101943 | LY 345899 | MTHFD |
| HY-101947 | SMI-16a | Pim |
| HY-101966 | NCT-503 | PHGDH |
| HY-101978 | CPI-444 | Adenosine Receptor |
| HY-101979A | Numidargistat (dihydrochloride) | Arginase |
| HY-101980 | Imaradenant | Adenosine Receptor |
| HY-102003 | Rucaparib (monocamsylate) | PARP |
| HY-102011 | BMS-1166 | PD-1/PD-L1 |
| HY-102031 | Linperlisib | PI3K |
| HY-102047B | KDOAM-25 (citrate) | Histone Demethylase |
| HY-102059 | MOZ-IN-2 | Histone Acetyltransferase |
| HY-102060 | WM-8014 | Histone Acetyltransferase |
| HY-102080 | SAFit2 | FKBP |
| HY-102087 | JPM-OEt | Cathepsin |
| HY-10249A | AKT Kinase Inhibitor | Akt |
| HY-103000 | HSF1A | HSP |
| HY-10303 | PIK-294 | PI3K |
| HY-103068 | Diethyl-pythiDC | MMP |
| HY-103085 | T-3775440 hydrochloride | Histone Demethylase |
| HY-103088 | Palupiprant | Prostaglandin Receptor |
| HY-103128 | inS3-54A18 | STAT |
| HY-10326 | GW788388 | TGF-β Receptor |
| HY-103380 | NSC 625987 | CDK |
| HY-103389 | 1-Aminobenzotriazole | Cytochrome P450 |
| HY-10343 | Sotrastaurin | PKC |
| HY-103444 | ARP-100 | MMP |
| HY-10346 | AV-412 | EGFR |
| HY-103482 | JNJ0966 | MMP |
| HY-103617 | PKM2-IN-1 | Pyruvate Kinase |
| HY-10366 | BI-78D3 | JNK |

| | | |
|---|---|---|
| HY-103667 | 2-HBA | Caspase |
| HY-103671 | IPN60090 | Glutaminase |
| HY-104010 | Asciminib | Bcr-Abl |
| HY-104048 | QC6352 | Histone Demethylase |
| HY-10406 | Talmapimod | p38 MAPK |
| HY-10502 | Tipifarnib | Farnesyl Transferase |
| HY-10505 | Orteronel | Cytochrome P450 |
| HY-10519 | BMS-345541 | IKK |
| HY-10528 | Tasquinimod | HDAC |
| HY-10550A | Tariquidar | P-glycoprotein |
| HY-10559 | Nelotanserin | 5-HT Receptor |
| HY-10619D | Niraparib (R-enantiomer) | PARP |
| HY-10627A | GW3965 (hydrochloride) | LXR |
| HY-10629 | LXR-623 | LXR |
| HY-106381 | Aurothiomalate (sodium) | PKC |
| HY-10680 | MK-6892 | GPR109A |
| HY-106994A | Nebentan (potassium) | Endothelin Receptor |
| HY-107146 | PZ-128 | Protease Activated Receptor (PAR) |
| HY-10721 | PF-AKT400 | Akt |
| HY-107361 | Golidocitinib | JAK |
| HY-107371 | 6-Bromo-2-hydroxy-3-methoxybenzaldehyde | IRE1 |
| HY-107385 | Epristeride | 5 alpha Reductase |
| HY-107397 | Ch55 | RAR/RXR |
| HY-107399 | CD3254 | RAR/RXR |
| HY-107412 | Proteasome inhibitor IX | Proteasome |
| HY-107424 | BAY-299 | Epigenetic Reader Domain |
| HY-107778 | PF-9366 | MAT |
| HY-107981 | LSN 3213128 | Antifolate |
| HY-107986 | GNE-6776 | Deubiquitinase |
| HY-107999 | CADD522 | Reactive Oxygen Species |
| HY-108314A | GC7 Sulfate | DNA/RNA Synthesis |
| HY-108360 | PDD 00017273 | PARG |
| HY-10858 | WAY 316606 | sFRP-1 |
| HY-108643 | CMPD1 | MAPKAPK2 (MK2) |
| HY-108691 | PF-06465469 | Itk |
| HY-108697 | PT2399 | HIF/HIF Prolyl-Hydroxylase |
| HY-108697A | (Rac)-PT2399 | HIF/HIF Prolyl-Hydroxylase |

| | | |
|---|---|---|
| HY-108708 | GeA-69 | PARP |
| HY-108886 | JWG-071 | ERK |
| HY-109080 | Belvarafenib | Raf |
| HY-109084 | Conteltinib | FAK |
| HY-109099 | Pemigatinib | FGFR |
| HY-109103 | Tegatrabetan | β-catenin |
| HY-109108 | Valemetostat | Histone Methyltransferase |
| HY-109115 | Fosifloxuridine nafalbenamide | Thymidylate Synthase |
| HY-109128 | Danicamtiv | Myosin |
| HY-10917 | GW2580 | c-Fms |
| HY-10929 | UNC0224 | Histone Methyltransferase |
| HY-10930 | UNC0321 | Histone Methyltransferase |
| HY-109566 | AZD1390 | ATM/ATR |
| HY-109574 | Raf inhibitor 2 | Raf |
| HY-109585 | IRAK4-IN-7 | IRAK |
| HY-10974 | MK-0752 | γ-secretase |
| HY-10977 | Tivozanib | VEGFR |
| HY-10993 | Adavosertib | Wee1 |
| HY-11000 | GSK429286A | ROCK |
| HY-11002 | CP-466722 | ATM/ATR |
| HY-11003 | GW843682X | Polo-like Kinase (PLK) |
| HY-110052 | TBCA | Casein Kinase |
| HY-11010 | AS601245 | JNK |
| HY-11012 | TDZD-8 | GSK-3 |
| HY-110193 | SPP-86 | RET |
| HY-110203 | R-7050 | TNF Receptor |
| HY-110266 | GN44028 | HIF/HIF Prolyl-Hydroxylase |
| HY-110287 | Apcin | APC |
| HY-11029 | SNT-207707 | Melanocortin Receptor |
| HY-110318 | VUF11207 (fumarate) | CXCR |
| HY-11035 | WAY-262611 | β-catenin |
| HY-110404 | Capzimin | Proteasome |
| HY-111050 | JNJ-38877618 | c-Met/HGFR |
| HY-111101 | AZ1495 | IRAK |
| HY-111102 | FL-411 | Epigenetic Reader Domain |
| HY-111108 | LDH-IN-1 | Lactate Dehydrogenase |
| HY-111109 | EZM 2302 | Histone Methyltransferase |

| | | |
|---|---|---|
| HY-111342 | HDAC8-IN-1 | HDAC |
| HY-111387 | IDF-11774 | HIF/HIF Prolyl-Hydroxylase |
| HY-111388A | SEL120-34A (monohydrochloride) | CDK |
| HY-111400 | SR-4370 | HDAC |
| HY-111472 | Porcn-IN-1 | Porcupine |
| HY-111482 | SM 16 | TGF-β Receptor |
| HY-111483 | Tizaterkib | ERK |
| HY-111498A | RGX-104 | LXR |
| HY-111507 | PDGFRα kinase inhibitor 1 | PDGFR |
| HY-111530 | GSTO1-IN-1 | Gutathione S-transferase |
| HY-111538 | MAGL-IN-1 | MAGL |
| HY-111558A | Bobcat339 (hydrochloride) | DNA Methyltransferase |
| HY-111752 | EML4-ALK kinase inhibitor 1 | ALK |
| HY-111905 | BRD7-IN-1 | Epigenetic Reader Domain |
| HY-111941 | GSK8612 | IKK |
| HY-111946 | GSK3145095 | RIP kinase |
| HY-112088 | AZD4573 | CDK |
| HY-112096 | eCF506 | Src |
| HY-112113 | SLV-2436 | MNK |
| HY-112130 | AGI-24512 | MAT |
| HY-112131 | MAT2A inhibitor 1 | MAT |
| HY-112140 | JH-VIII-157-02 | ALK |
| HY-112167A | GDC-0575 dihydrochloride | Checkpoint Kinase (Chk) |
| HY-112181 | KO-947 | ERK |
| HY-112218 | MIK665 | Bcl-2 Family |
| HY-112261 | CDK12-IN-3 | CDK |
| HY-112301A | trans-Pralsetinib | RET |
| HY-112420 | EGFR/ErbB-2/ErbB-4 inhibitor-2 | EGFR |
| HY-112424 | ETC-206 | MNK |
| HY-112427 | Sirt2-IN-1 | Sirtuin |
| HY-112429 | HJB97 | Epigenetic Reader Domain |
| HY-112438 | MF-094 | Deubiquitinase |
| HY-112443 | AZD3458 | PI3K |
| HY-112445 | SGC3027 | Histone Methyltransferase |
| HY-112534 | GSTO-IN-2 | Gutathione S-transferase |
| HY-112569 | MAT2A inhibitor 2 | MAT |
| HY-112701 | CCR6 inhibitor 1 | CCR |

| | | |
|---|---|---|
| HY-112719 | BRD 4354 | HDAC |
| HY-112734 | 4'-Methylchrysoeriol | Cytochrome P450 |
| HY-112802A | AZD3229 Tosylate | c-Kit |
| HY-112815 | ALK2-IN-2 | TGF-β Receptor |
| HY-112823 | Almonertinib | EGFR |
| HY-112829 | Denifanstat | Fatty Acid Synthase (FASN) |
| HY-112859 | VU0661013 | Bcl-2 Family |
| HY-114169 | WRG-28 | Discoidin Domain Receptor |
| HY-114189 | GW284543 | MEK |
| HY-114208A | BI-9321 (trihydrochloride) | Histone Methyltransferase |
| HY-114209 | MRK-740 | Histone Methyltransferase |
| HY-114258 | LY3295668 | Aurora Kinase |
| HY-114263 | NXT629 | PPAR |
| HY-114277 | Sotorasib | Ras |
| HY-114302 | CCB02 | Microtubule/Tubulin |
| HY-114339 | CDK4/6-IN-2 | CDK |
| HY-114370 | Selpercatinib | RET |
| HY-114409 | GB1107 | Galectin |
| HY-114410 | CCI-006 | Mitochondrial Metabolism |
| HY-114436 | MRTX-1257 | Ras |
| HY-114454 | INH14 | IKK |
| HY-114657A | Benproperine (phosphate) | Arp2/3 Complex |
| HY-115487 | MF-766 | Prostaglandin Receptor |
| HY-115677 | ILK-IN-3 | Integrin |
| HY-115686 | 8-Azaadenosine | Adenosine Deaminase |
| HY-116761 | GSK467 | Histone Demethylase |
| HY-116856 | SRPKIN-1 | SRPK |
| HY-117240 | NCT-502 | PHGDH |
| HY-117288A | S55746 (hydrochloride) | Bcl-2 Family |
| HY-117407 | ALLO-2 | Smo |
| HY-117535 | CDK2-IN-4 | CDK |
| HY-117596 | UNC569 | TAM Receptor |
| HY-117793 | I-191 | PAR |
| HY-119016A | SK1-?I (hydrochloride) | SphK |
| HY-119039 | RU-301 | TAM Receptor |
| HY-119377 | UPGL00004 | Glutaminase |
| HY-119939 | CHDI-390576 | HDAC |

| | | |
|---|---|---|
| HY-119940 | MC180295 | CDK |
| HY-12001 | WZ-3146 | EGFR |
| HY-120145 | MST-312 | Telomerase |
| HY-12017 | PF-04217903 | c-Met/HGFR |
| HY-120204 | BI8626 | E1/E2/E3 Enzyme |
| HY-12026 | WZ4002 | EGFR |
| HY-120350 | BI-1347 | CDK |
| HY-12042 | Pimasertib | MEK |
| HY-12050 | CP-673451 | PDGFR |
| HY-120528A | GB-110 (hydrochloride) | PAR |
| HY-12058 | AZD8330 | MEK |
| HY-12060 | KRN-633 | VEGFR |
| HY-12062 | PD318088 | MEK |
| HY-12068 | PI3K-IN-1 | PI3K |
| HY-12071A | LDN193189 (Tetrahydrochloride) | TGF-β Receptor |
| HY-120722 | TCH-165 | Proteasome |
| HY-120856 | ARN-3236 | Salt-inducible Kinase (SIK) |
| HY-120929 | BI8622 | E1/E2/E3 Enzyme |
| HY-121167 | L-Albizziin | Glutaminase |
| HY-12136 | Purpurogallin | Xanthine Oxidase |
| HY-12169 | Marimastat | MMP |
| HY-12202 | MEK inhibitor | MEK |
| HY-122022 | JR-AB2-011 | mTOR |
| HY-122181B | OTS186935 (hydrochloride) | Histone Methyltransferase |
| HY-122186 | SGC-GAK-1 | Cyclin G-associated Kinase (GAK) |
| HY-122197 | ML339 | CXCR |
| HY-12220A | MM-102 (TFA) | Histone Methyltransferase |
| HY-122312 | BAY-8002 | Monocarboxylate Transporter |
| HY-12235 | PI-3065 | PI3K |
| HY-12239 | CID755673 | PKD |
| HY-12241 | AZ82 | Kinesin |
| HY-122590 | Glabrescione B | Gli |
| HY-122632 | Ciliobrevin D | Hedgehog |
| HY-122641B | Deltasonamide 2 (TFA) | Phosphodiesterase (PDE) |
| HY-122705 | RAD51-IN-1 | RAD51 |
| HY-12276 | MALT1 inhibitor MI-2 | MALT1 |
| HY-12277 | AZ191 | DYRK |

| | | |
|---|---|---|
| HY-12285 | Serabelisib | PI3K |
| HY-122856 | AZ12601011 | TGF-β Receptor |
| HY-122881 | HOIPIN-1 | IKK |
| HY-12289 | Defactinib | FAK |
| HY-122903 | TK216 | DNA/RNA Synthesis |
| HY-12291 | HG6-64-1 | Raf |
| HY-123242 | FTI-2153 | Farnesyl Transferase |
| HY-12325 | GSK2194069 | Fatty Acid Synthase (FASN) |
| HY-12330 | AZD8186 | PI3K |
| HY-12334 | HTH-01-015 | AMPK |
| HY-12335 | UNC0379 | Histone Methyltransferase |
| HY-12340 | ETP-46321 | PI3K |
| HY-12354 | SB-3CT | MMP |
| HY-12358 | Tpl2 Kinase Inhibitor 1 | MAP3K |
| HY-123593 | Mozavaptan (hydrochloride) | Vasopressin Receptor |
| HY-12365 | Namodenoson | Adenosine Receptor |
| HY-123772 | CDK5 inhibitor 20-223 | CDK |
| HY-12382 | NMS-P715 | Mps1 |
| HY-123892 | RK-287107 | PARP |
| HY-123918 | JMS-17-2 | CX3CR1 |
| HY-12409 | PFI-3 | Epigenetic Reader Domain |
| HY-12412 | ML132 | Caspase |
| HY-12418 | E7449 | PARP |
| HY-12438 | G007-LK | PARP |
| HY-12444 | Y15 | FAK |
| HY-124447 | BTYNB | c-Myc |
| HY-124573 | OBI-3424 | DNA Alkylator/Crosslinker |
| HY-124628 | IPI-9119 | Fatty Acid Synthase (FASN) |
| HY-124653 | HSP27 inhibitor J2 | HSP |
| HY-12466 | Z-DEVD-FMK | Caspase |
| HY-124798 | Rheb inhibitor NR1 | mTOR |
| HY-12492 | VER-246608 | PDHK |
| HY-12493A | LY-2584702 (tosylate salt) | Ribosomal S6 Kinase (RSK) |
| HY-12494 | LDC1267 | TAM Receptor |
| HY-125166 | DB04760 | MMP |
| HY-125286 | AB-680 | CD73 |
| HY-125837A | MS31 (trihydrochloride) | Epigenetic Reader Domain |

| | | |
|---|---|---|
| HY-125840 | Belzutifan | HIF/HIF Prolyl-Hydroxylase |
| HY-125974 | HM03 | HSP |
| HY-126073 | DFP00173 | Aquaporin |
| HY-126146 | PKC-iota inhibitor 1 | PKC |
| HY-12622 | HSP70-IN-1 | HSP |
| HY-126246 | CDC25B-IN-1 | Phosphatase |
| HY-126254 | BI-4924 | PHGDH |
| HY-12628 | GNE-618 | NAMPT |
| HY-126297 | c-Fms-IN-10 | c-Fms |
| HY-12644 | Acalisib | PI3K |
| HY-12682 | Glutaminase C-IN-1 | Glutaminase |
| HY-12683 | BPTES | Glutaminase |
| HY-127104 | FMF-04-159-2 | CDK |
| HY-12746 | DC-05 | DNA Methyltransferase |
| HY-12747 | DC_517 | DNA Methyltransferase |
| HY-12750 | AZD3965 | Monocarboxylate Transporter |
| HY-12752A | Alimemazine hemitartrate | Histamine Receptor |
| HY-12754 | ML228 | HIF/HIF Prolyl-Hydroxylase |
| HY-12757 | YHO-13177 | BCRP |
| HY-12759 | CARM1-IN-1 | Histone Methyltransferase |
| HY-12782T | Iadademstat (dihydrochloride) | Histone Demethylase |
| HY-12812 | Autotaxin modulator 1 | Phosphodiesterase (PDE) |
| HY-12814 | TH588 | DNA/RNA Synthesis |
| HY-12823 | BLU9931 | FGFR |
| HY-12830 | M-110 | Pim |
| HY-128341 | ERK5-IN-2 | ERK |
| HY-12838 | Mirk-IN-1 | DYRK |
| HY-12848 | SAG | Smo |
| HY-12857 | Brigatinib | ALK |
| HY-128586A | TAS4464 (hydrochloride) | NEDD8-activating Enzyme |
| HY-12861 | CB-5083 | p97 |
| HY-12879 | IWP-4 | Wnt |
| HY-12948 | AMG319 | PI3K |
| HY-129490 | Enpp-1-IN-1 | Phosphodiesterase (PDE) |
| HY-129545 | DS-1001b | Isocitrate Dehydrogenase (IDH) |
| HY-12964 | SGI-7079 | TAM Receptor |
| HY-12974 | PRT-060318 | Syk |

| | | |
|---|---|---|
| HY-12975 | AZ6102 | PARP |
| HY-13011 | Alectinib | ALK |
| HY-130208 | Thiodigalactoside | Galectin |
| HY-13022 | CC-401 (hydrochloride) | JNK |
| HY-130240 | GCN2-IN-6 | Eukaryotic Initiation Factor (eIF) |
| HY-130251 | DS18561882 | MTHFD |
| HY-13050 | Sapitinib | EGFR |
| HY-130538 | 1-Naphthohydroxamic acid | HDAC |
| HY-13055 | Telotristat etiprate | Tryptophan Hydroxylase |
| HY-13057 | O6BTG-octylglucoside | DNA Methyltransferase |
| HY-13078 | Cobimetinib (racemate) | MEK |
| HY-131003 | Taletrectinib | ROS Kinase |
| HY-131066 | EMI48 | EGFR |
| HY-131067 | EMI56 | EGFR |
| HY-131339 | SP-96 | Aurora Kinase |
| HY-131341 | Syk-IN-4 | Syk |
| HY-131345 | PI3Kα-IN-4 | PI3K |
| HY-13215 | Avasimibe | Acyltransferase |
| HY-132166 | M4205 | c-Kit |
| HY-132172 | TAK1-IN-2 | MAP3K |
| HY-132175 | CK2 inhibitor 2 | Casein Kinase |
| HY-132192 | PD-1/PD-L1-IN-9 | PD-1/PD-L1 |
| HY-13222 | BAN ORL 24 | Opioid Receptor |
| HY-13226 | Galunisertib | TGF-β Receptor |
| HY-13227 | SD-208 | TGF-β Receptor |
| HY-132283 | PF-9363 | Histone Acetyltransferase |
| HY-13254 | A-674563 | Akt |
| HY-13255A | TAME hydrochloride | APC |
| HY-13258 | NVP-BHG712 isomer | Ephrin Receptor |
| HY-13258A | NVP-BHG712 | Ephrin Receptor |
| HY-13261 | A66 | PI3K |
| HY-13266A | BS-181 (hydrochloride) | CDK |
| HY-13282 | GANT 58 | Gli |
| HY-13298 | Mps1-IN-1 | Mps1 |
| HY-13304 | LY2874455 | FGFR |
| HY-13307 | JK184 | Hedgehog |
| HY-133083 | BAY-474 | c-Met/HGFR |

| | | |
|---|---|---|
| HY-133117 | BAY-985 | IKK |
| HY-13319 | JNK-IN-8 | JNK |
| HY-13330 | AZD4547 | FGFR |
| HY-133511 | MLS000544460 | Phosphatase |
| HY-133907 | NVS-PI3-4 | PI3K |
| HY-13427 | Allitinib tosylate | EGFR |
| HY-13449 | TAK-733 | MEK |
| HY-13463B | Avatrombopag (hydrochloride) | Thrombopoietin Receptor |
| HY-13470 | GSK126 | Histone Methyltransferase |
| HY-134828 | AZ506 | Histone Methyltransferase |
| HY-13487 | USP7/USP47 inhibitor | Deubiquitinase |
| HY-13491 | GNF-5837 | Trk Receptor |
| HY-134957 | VT107 | YAP |
| HY-135127 | Dot1L-IN-4 | Histone Methyltransferase |
| HY-13513 | U-104 | Carbonic Anhydrase |
| HY-135146 | GSK-3484862 | DNA Methyltransferase |
| HY-135232 | MMP-9-IN-1 | MMP |
| HY-135236 | OXFBD04 | Epigenetic Reader Domain |
| HY-13535A | ATN-161 (trifluoroacetate salt) | Integrin |
| HY-13537A | BPR1J-097 Hydrochloride | FLT3 |
| HY-135516 | Wnt pathway activator 1 | Wnt |
| HY-135815 | Mobocertinib | EGFR |
| HY-13593 | Chlorambucil | DNA Alkylator/Crosslinker |
| HY-13600 | Clobetasol propionate | Cytochrome P450 |
| HY-136174 | RBN-2397 | PARP |
| HY-136175 | Revumenib | Epigenetic Reader Domain |
| HY-136244 | PF-06952229 | TGF-β Receptor |
| HY-136254 | BzATP (triethylammonium salt) | P2X Receptor |
| HY-136270 | Gartisertib | ATM/ATR |
| HY-136328 | EZM0414 (TFA) | Histone Methyltransferase |
| HY-13634B | TLK117 | Gutathione S-transferase |
| HY-13635 | Finasteride | 5 alpha Reductase |
| HY-136350 | BRD9500 | Phosphodiesterase (PDE) |
| HY-136360 | MI-3454 | Epigenetic Reader Domain |
| HY-13643 | Daminozide | Histone Demethylase |
| HY-136464 | β-catenin-IN-2 | β-catenin |
| HY-13646A | Encequidar (mesylate) | P-glycoprotein |

| | | |
|---|---|---|
| HY-13650 | Indisulam | Carbonic Anhydrase |
| HY-136530 | SR18662 | KLF |
| HY-136538 | LQZ-7I | Survivin |
| HY-136546 | Stafia-1 | STAT |
| HY-136567 | TBAP-001 | Raf |
| HY-136579 | ERK-IN-3 | ERK |
| HY-13668 | Lomeguatrib | DNA Methyltransferase |
| HY-136684 | BrBzGCp2 | Glyoxalase (GLO) |
| HY-13688 | PJ34 (hydrochloride) | PARP |
| HY-136895 | AZ12672857 | Prostaglandin Receptor |
| HY-136978 | OP-5244 | CD73 |
| HY-136979 | RBN012759 | PARP |
| HY-137067 | IMT1B | DNA/RNA Synthesis |
| HY-137187 | FB23 | FTO |
| HY-137460 | Vodobatinib | Bcr-Abl |
| HY-13761 | Teniposide | Topoisomerase |
| HY-13773 | Motolimod | Toll-like Receptor (TLR) |
| HY-138001 | WH-4-025 | Salt-inducible Kinase (SIK) |
| HY-13802 | SC-514 | IKK |
| HY-13807 | UNC0646 | Histone Methyltransferase |
| HY-138239 | WEE1-IN-3 | Wee1 |
| HY-13831 | BPTU | P2Y Receptor |
| HY-138537 | NF-κB-IN-1 | IKK |
| HY-138565 | K-975 | YAP |
| HY-138630 | AG-270 | MAT |
| HY-13867 | Bisindolylmaleimide I | PKC |
| HY-138742 | HPK1-IN-7 | MAP4K |
| HY-138751 | limertinib | EGFR |
| HY-138794 | XL177A | Deubiquitinase |
| HY-13897 | CNX-2006 | EGFR |
| HY-13907 | TCS 359 | FLT3 |
| HY-13909 | RGFP966 | HDAC |
| HY-139139 | MAT2A inhibitor 3 | MAT |
| HY-139536 | MAT2A inhibitor 4 | MAT |
| HY-13979 | DDR1-IN-1 | Discoidin Domain Receptor |
| HY-13982 | JSH-23 | NF-κB |
| HY-13983 | IDO-IN-7 | IDO |

| | | |
|---|---|---|
| HY-13984 | Mutant EGFR inhibitor | EGFR |
| HY-141659 | USP30 inhibitor 18 | Deubiquitinase |
| HY-141716 | SW2_110A | Histone Methyltransferase |
| HY-14177 | Raf inhibitor 1 | Raf |
| HY-14258A | Escitalopram (oxalate) | Serotonin Transporter |
| HY-14280 | Entacapone | COMT |
| HY-14369 | Elagolix sodium | GnRH Receptor |
| HY-14463 | Onalespib | HSP |
| HY-14483 | AF-353 | P2X Receptor |
| HY-14530 | Pelitrexol | Antifolate |
| HY-14566 | Donepezil | Cholinesterase (ChE) |
| HY-14592 | Tectochrysin | NF-κB |
| HY-14617 | Paradol | COX |
| HY-14645 | (-)-DHMEQ | NF-κB |
| HY-14691 | Refametinib | MEK |
| HY-14715B | CCT241533 (hydrochloride) | Checkpoint Kinase (Chk) |
| HY-14719 | RO4987655 | MEK |
| HY-14731 | VE-821 | ATM/ATR |
| HY-14734 | Anamorelin | GHSR |
| HY-14761 | Bentamapimod | JNK |
| HY-14807 | Tosedostat | Aminopeptidase |
| HY-14846 | Litronesib | Kinesin |
| HY-14909 | Bardoxolone | Keap1-Nrf2 |
| HY-14927 | Lificiguat | Guanylate Cyclase |
| HY-14950 | Dalcetrapib | CETP |
| HY-15004 | AUZ 454 | CDK |
| HY-15102 | MK-0429 | Integrin |
| HY-15150 | Bemcentinib | TAM Receptor |
| HY-15164 | Icotinib (Hydrochloride) | EGFR |
| HY-15167A | Glyoxalase I inhibitor (free base) | Glyoxalase (GLO) |
| HY-15186 | Ipatasertib | Akt |
| HY-15191 | Sabutoclax | Bcl-2 Family |
| HY-15193 | EMD638683 | SGK |
| HY-15217 | CHR-6494 | Haspin Kinase |
| HY-15226A | AZ505 (ditrifluoroacetate) | Histone Methyltransferase |
| HY-15237 | SL 0101-1 | Ribosomal S6 Kinase (RSK) |
| HY-15241 | NVP-LCQ195 | CDK |

| | | |
|---|---|---|
| HY-15244 | Alpelisib | PI3K |
| HY-15251 | Reparixin | CXCR |
| HY-15260A | XL413 (hydrochloride) | CDK |
| HY-15272 | WAY-600 | mTOR |
| HY-15283 | Clopidogrel | P2Y Receptor |
| HY-15290 | AIM-100 | Ack1 |
| HY-15294 | CZC24832 | PI3K |
| HY-15307 | Belumosudil | ROCK |
| HY-15321 | Etoricoxib | COX |
| HY-15338 | TG003 | CDK |
| HY-15341 | BAM7 | Bcl-2 Family |
| HY-15424 | 5-Iodotubercidin | Adenosine Kinase |
| HY-15437 | SL327 | MEK |
| HY-15452A | (S)-Selisistat | Sirtuin |
| HY-15458 | SAR131675 | VEGFR |
| HY-15466 | Izorlisib | PI3K |
| HY-15467A | ZM323881 (hydrochloride) | VEGFR |
| HY-15478 | WZ811 | CXCR |
| HY-15505 | RWJ-67657 | p38 MAPK |
| HY-15508 | JANEX-1 | JAK |
| HY-15512A | OTSSP167 (hydrochloride) | MELK |
| HY-15513 | TC-DAPK 6 | DAPK |
| HY-15532 | SCH900776 | Checkpoint Kinase (Chk) |
| HY-15542B | FRAX486 | PAK |
| HY-15590 | AZ-23 | Trk Receptor |
| HY-15599 | SSR128129E | FGFR |
| HY-15607A | WEHI-539 hydrochloride | Bcl-2 Family |
| HY-15650 | SGC0946 | Histone Methyltransferase |
| HY-15663 | IPA-3 | PAK |
| HY-15679 | PI4KIII beta inhibitor 3 | PI4K |
| HY-15681 | Senexin A | CDK |
| HY-15689 | Epacadostat | IDO |
| HY-15698A | CRT0066101 dihydrochloride | PKD |
| HY-15713 | NMS-873 | p97 |
| HY-15729 | Rociletinib | EGFR |
| HY-15744 | LY255283 | Leukotriene Receptor |
| HY-15754 | CGP37157 | Na+/Ca2+ Exchanger |

| | | |
|---|---|---|
| HY-15757 | SJB2-043 | Deubiquitinase |
| HY-15768 | Ilomastat | MMP |
| HY-15772 | Osimertinib | EGFR |
| HY-15775 | Arginase inhibitor 1 | Arginase |
| HY-15797 | UNC2250 | TAM Receptor |
| HY-15802 | WZ4003 | AMPK |
| HY-15816 | Ulixertinib | ERK |
| HY-15825 | IWP L6 | Porcupine |
| HY-15837 | SAR-260301 | PI3K |
| HY-15838 | ID-8 | DYRK |
| HY-15845 | STF-083010 | IRE1 |
| HY-15857 | CW-069 | Kinesin |
| HY-15877 | BTB06584 | ATP Synthase |
| HY-15890 | C-7280948 | Histone Methyltransferase |
| HY-15892 | CK-636 | Arp2/3 Complex |
| HY-15948 | Kif15-IN-1 | Kinesin |
| HY-15949 | Kif15-IN-2 | Kinesin |
| HY-15958 | VBY-825 | Cathepsin |
| HY-15959 | Savolitinib | c-Met/HGFR |
| HY-15965 | Uprosertib | Akt |
| HY-15996 | Seviteronel | Cytochrome P450 |
| HY-16014 | A-385358 | Bcl-2 Family |
| HY-16122B | CAL-130 (Hydrochloride) | PI3K |
| HY-16273A | L-778123 (hydrochloride) | Farnesyl Transferase |
| HY-16294 | LY2090314 | GSK-3 |
| HY-16297 | Abemaciclib (methanesulfonate) | CDK |
| HY-16355 | NVP-QAV-572 | PI3K |
| HY-16381A | Pasireotide (acetate) | Somatostatin Receptor |
| HY-16391 | Glasdegib | Smo |
| HY-16500 | Tolrestat | Aldose Reductase |
| HY-16510 | UNC926 | Epigenetic Reader Domain |
| HY-16666 | 3CAI | Akt |
| HY-16697 | CID 16020046 | GPR55 |
| HY-16706A | Remodelin (hydrobromide) | Histone Acetyltransferase |
| HY-16724 | Indoximod | IDO |
| HY-16927 | CK-869 | Arp2/3 Complex |
| HY-16965 | TH287 | DNA/RNA Synthesis |

| | | |
|---|---|---|
| HY-16972 | SR9243 | LXR |
| HY-16975 | SH-4-54 | STAT |
| HY-16976 | GDC-0339 | Pim |
| HY-16988 | SR9011 | REV-ERB |
| HY-16997 | Itacitinib | JAK |
| HY-17034A | Dexmedetomidine (hydrochloride) | Adrenergic Receptor |
| HY-17042A | Cetirizine (dihydrochloride) | Histamine Receptor |
| HY-17044 | Duvelisib | PI3K |
| HY-17372 | Rofecoxib | COX |
| HY-17498 | Atenolol | Adrenergic Receptor |
| HY-18007 | ALW-II-41-27 | Ephrin Receptor |
| HY-18030A | CEP-28122 (mesylate salt) | ALK |
| HY-18075 | LPA2 antagonist 1 | LPL Receptor |
| HY-18086 | TCS PIM-1 1 | Pim |
| HY-18095 | CX-6258 | Pim |
| HY-18304 | BMS-3 | LIM Kinase (LIMK) |
| HY-18305 | BMS-5 | LIM Kinase (LIMK) |
| HY-18342 | Diflunisal | COX |
| HY-18360 | TMP269 | HDAC |
| HY-18361 | TMP195 | HDAC |
| HY-18602 | FIIN-2 | FGFR |
| HY-18609 | PD158780 | EGFR |
| HY-18627A | PFI-2 (hydrochloride) | Histone Methyltransferase |
| HY-18657 | TEPP-46 | Pyruvate Kinase |
| HY-18662 | RQ-00203078 | TRP Channel |
| HY-18664 | PFI-4 | Epigenetic Reader Domain |
| HY-18665 | GSK-5959 | Epigenetic Reader Domain |
| HY-18682 | Centrinone | Polo-like Kinase (PLK) |
| HY-18683 | Centrinone-B | Polo-like Kinase (PLK) |
| HY-18717 | Mutant IDH1-IN-2 | Isocitrate Dehydrogenase (IDH) |
| HY-18731 | 1400W (Dihydrochloride) | NO Synthase |
| HY-18744 | FLT3-IN-2 | FLT3 |
| HY-18750 | Zorifertinib | EGFR |
| HY-18758 | IN-1130 | TGF-β Receptor |
| HY-18768 | NCT-501 | ALDH |
| HY-18770A | IDO-IN-6 | IDO |
| HY-18777 | KC7F2 | HIF/HIF Prolyl-Hydroxylase |

| | | |
|---|---|---|
| HY-18785 | Indirubin Derivative E804 | IGF-1R |
| HY-18937 | WEHI-345 | RIP kinase |
| HY-18958 | CCT245737 | Checkpoint Kinase (Chk) |
| HY-18971 | TG4-155 | Prostaglandin Receptor |
| HY-18975 | I-BRD9 | Epigenetic Reader Domain |
| HY-18998 | LMK-235 | HDAC |
| HY-19121A | TCV-309 (chloride) | PAFR |
| HY-19313 | LLY-507 | Histone Methyltransferase |
| HY-19336 | BAZ2-ICR | Epigenetic Reader Domain |
| HY-19340 | TMS | Cytochrome P450 |
| HY-19347 | WDR5-0103 | Histone Methyltransferase |
| HY-19352 | T56-LIMKi | LIM Kinase (LIMK) |
| HY-19387 | Didox | DNA/RNA Synthesis |
| HY-19530 | PF-2771 | Kinesin |
| HY-19535A | Nemiralisib | PI3K |
| HY-19546 | BAY-598 | Histone Methyltransferase |
| HY-19612B | DDP-38003 (trihydrochloride) | Histone Demethylase |
| HY-19626 | NSC23925 | P-glycoprotein |
| HY-19635 | G-5555 | PAK |
| HY-19702 | PKR-IN-2 | Pyruvate Kinase |
| HY-19713 | LJI308 | Ribosomal S6 Kinase (RSK) |
| HY-19737A | DG172 (dihydrochloride) | PPAR |
| HY-19741 | A-1331852 | Bcl-2 Family |
| HY-19745A | N-deacetylated BMS-202 | PD-1/PD-L1 |
| HY-19753 | KS176 | BCRP |
| HY-19756 | OTX008 | Galectin |
| HY-19763 | BEBT-908 | PI3K |
| HY-19794 | MP-A08 | SphK |
| HY-19797A | ML241 (hydrochloride) | p97 |
| HY-19803 | Naquotinib (mesylate) | EGFR |
| HY-19928 | Vactosertib | TGF-β Receptor |
| HY-19931 | COH29 | DNA/RNA Synthesis |
| HY-19957 | Zoligratinib | FGFR |
| HY-19981 | Derazantinib | FGFR |
| HY-19983 | ASP5878 | FGFR |
| HY-19994 | ML264 | KLF |
| HY-21972 | BCI-121 | Histone Methyltransferase |

| | | |
|---|---|---|
| HY-30237 | Seliciclib | CDK |
| HY-32018 | Cot inhibitor-2 | MAP3K |
| HY-50098A | Mardepodect (hydrochloride) | Phosphodiesterase (PDE) |
| HY-50667 | Apixaban | Factor Xa |
| HY-50710 | KU-0063794 | mTOR |
| HY-50846 | SCH772984 | ERK |
| HY-50864 | GDC-0879 | Raf |
| HY-50903 | Rivaroxaban | Factor Xa |
| HY-51424 | PLX-4720 | Raf |
| HY-52101 | CMK | Ribosomal S6 Kinase (RSK) |
| HY-52101A | FMK | Ribosomal S6 Kinase (RSK) |
| HY-70062 | Pevonedistat | NEDD8-activating Enzyme |
| HY-70072 | D609 | Phospholipase |
| HY-70074 | CCG-63802 | RGS Protein |
| HY-76251 | Etodolac | COX |
| HY-76316 | Bergaptol | Cytochrome P450 |
| HY-79583 | Glutaminase-IN-3 | Glutaminase |
| HY-80013 | THZ1 | CDK |
| HY-A0014 | Ramelteon | Melatonin Receptor |
| HY-A0060 | Malotilate | Lipoxygenase |
| HY-A0261 | Pentagastrin | Cholecystokinin Receptor |
| HY-B0189A | Mosapride (citrate) | 5-HT Receptor |
| HY-B0219 | Allopurinol | Xanthine Oxidase |
| HY-B0305A | Roxatidine (Acetate Hydrochloride) | Histamine Receptor |
| HY-B0310 | Nizatidine | Histamine Receptor |
| HY-B0331A | Enalapril (maleate) | ACE |
| HY-B0375A | Argatroban (monohydrate) | Thrombin |
| HY-B0744D | L-Eflornithine (monohydrochloride) | Parasite |
| HY-B1581A | L-Canavanine sulfate | NO Synthase |
| HY-B2147 | 2-Aminobenzenesulfonamide | Carbonic Anhydrase |
| HY-D0067 | 7ACC1 | Monocarboxylate Transporter |
| HY-N0085 | Dimethylfraxetin | Carbonic Anhydrase |
| HY-N0119 | Naringin Dihydrochalcone | NF-κB |
| HY-N0125 | Diosmetin | Cytochrome P450 |
| HY-N0139 | Troxerutin | NOD-like Receptor (NLR) |
| HY-N0319 | Salvianolic acid C | Cytochrome P450 |
| HY-N0628 | Kaempferitrin | Insulin Receptor |

| | | |
|---|---|---|
| HY-N0931 | Santacruzamate A | HDAC |
| HY-N1775 | 3',4'-Dihydroxyacetophenone | Tyrosinase |
| HY-N2022 | Castanospermine | Glucosidase |
| HY-N2194 | Bergamottin | Cytochrome P450 |
| HY-N2259 | Curcumenol | Cytochrome P450 |
| HY-N2262 | 8-Geranyloxypsoralen | Cytochrome P450 |
| HY-N2357 | Eudesmin | Ribosomal S6 Kinase (RSK) |
| HY-N2523 | Gigantol | Wnt |
| HY-N2600 | Kuwanon H | Bombesin Receptor |
| HY-N4149 | Quercetagetin | Pim |
| HY-N4288 | 4-Methylesculetin | Glutathione Peroxidase |
| HY-N4289 | 3-Epiursolic Acid | Cathepsin |
| HY-N5011 | 5,7-Dimethoxyflavone | Cytochrome P450 |
| HY-N6596 | 7-Hydroxy-4H-chromen-4-one | Src |
| HY-N6775 | Sonolisib | PI3K |
| HY-N6884 | Bixin | Reactive Oxygen Species |
| HY-N7368 | Hibifolin | Adenosine Deaminase |
| HY-P0299A | TSP-1 | TGF-β Receptor |
| HY-P1043A | NGR peptide (Trifluoroacetate) | Aminopeptidase |
| HY-P1187 | HSDVHK-NH2 | Integrin |
| HY-P1423A | BA 1 (TFA) | Bombesin Receptor |
| HY-P1545A | ACTH (1-17) (TFA) | Melanocortin Receptor |
| HY-P2230 | Angstrom6 | PAI-1 |
| HY-P3124A | BIM-23190 (hydrochloride) | Somatostatin Receptor |
| HY-U00177 | GDP366 | Survivin |
| HY-U00418 | ARS-1620 | Ras |
| HY-U00428A | GNE 220 (hydrochloride) | MAP4K |
| HY-U00435 | Neoseptin 3 | Toll-like Receptor (TLR) |
| HY-U00458 | K-80003 | Akt |
| HY-U00459 | GSK2850163 | IRE1 |
| HY-W011266 | JNJ-10198409 | PDGFR |
| HY-W014622 | CRT0044876 | DNA/RNA Synthesis |
| HY-W018781 | Benzamidine (hydrochloride) | Ser/Thr Protease |
| HY-W044764 | 2-Benzylsuccinic acid | Carboxypeptidase |
| HY-X0150 | JSH-150 | CDK |

**Table S2.** List of primers used in this study.

| Primers for real-time PCR | Primer sequence (5'-3') |
|---|---|
| *c-Myc* | F: GGCTCCTGGCAAAAGGTCA<br>R: CTGCGTAGTTGTGCTGATGT |
| *CARM1* | F: TCGCCACACCCAACGATTT<br>R: GTACTGCACGGCAGAAGACT |
| *CAD* | F: AGTGGTGTTTCAAACCGGCAT<br>R: CAGAGGATAGGTGAGCACTAAGA |
| *DHODH* | F: CCACGGGAGATGAGCGTTTC<br>R: CAGGGAGGTGAAGCGAACA |
| *UMPS* | F: TCTCGACCGCGTCTTCTGA<br>R: ACACACGGTGTCAAAACTGAT |
| *ACSL4* | F: CATCCCTGGAGCAGATACTCT<br>R: TCACTTAGGATTTCCCTGGTCC |
| *PLAA* | F: ACCTTGCAGGGTCATACAGC<br>R: CCAGCCTTCCACAGTTTAACAG |
| $PLA_2$ | F: TACCAGCACATTATAGTGGAGCA<br>R: GCTGTCAGGGGTTGTAGAGAT |
| *5-LOX* | F: ACTGGCTGAATGACGACTGG<br>R: CAGGGGAACTCGATGTAGTCC |
| *GPX4* | F: GAGGCAAGACCGAAGTAAACTAC<br>R: CCGAACTGGTTACACGGGAA |
| *GAPDH* | F: GGAGCGAGATCCCTCCAAAAT<br>R: GGCTGTTGTCATACTTCTCATGG |
| **Primers for ChIP** | **Primer sequence (5'-3')** |
| ChIP-ACSL4 | F: AGAGGAGCAGTGAAACCCAAA<br>R: CGATCCGCTTCTGTCAGTCT |
| ChIP-$PLA_2$ | F: AGGTGTTCTGAGTCTGGAGTG<br>R: ATGAGTGCTGTTGTGGATTTCT |
| ChIP-CAD | F: GAGAGCCACAAGACCAGGAG<br>R: AGCAGAATGGAGACGGATAGAG |
| ChIP-DHODH | F: TTCTCCAGGAGGCACAACAG<br>R: GAGGCAGGCAGATCACTTGA |
| ChIP-UMPS | F: ATCTTCCTGTTCCTGGCTTGT<br>R: GTCTGTACTCTGCTTCCTGGA |
| **shRNA sequences** | **Primer sequence (5'-3')** |
| shNC | AGTCTTAATCGCGTATAAGGC |
| shc-Myc-1 | GGAAACGACGAGAACAGTTGA |
| shc-Myc-2 | CCTGAGACAGATCAGCAACAA |
| shCARM1-1 | CTATGACTTGAGCAGTGTTAT |
| shCARM1-2 | CGATTTCTGTTCCTTCTACAA |
| **sgRNA sequences** | **Primer sequence (5'-3')** |
| sgNC | ACGGAGGCTAAGCGTCGCAA |
| sgc-Myc-1 | TGCTCGCCCTCCTACGTTG |

| | |
|---|---|
| sgc-Myc-2 | CCCTTCGGGGAGACAACGA |
| sgc-Myc-3 | GATGAAGGTCTCGTCGTCC |
| **siRNA sequences** | **Primer sequence (5'-3')** |
| sic-Myc-1 | F: GCUUCACCAACAGGAACUATT<br>R: UAGUUCCUGUUGGUGAAGCTT |
| sic-Myc-2 | F: GGCGAACACACAACGUCUUTT<br>R: AAGACGUUGUGUGUUCGCCTT |
| sic-Myc-3 | F: GGAAGAAAUCGAUGUUGUUTT<br>R: AACAACAUCGAUUUCUUCCTT |

**Table S3.** List of antibodies used in this study.

| Antibody | Source | Identifier |
|---|---|---|
| β-actin | Cell Signaling Technology | #4970 |
| c-Myc | Cell Signaling Technology | #9402 |
| c-Myc | Abcam | ab32072 |
| CARM1 | Abcam | ab243638 |
| P300 | Cell Signaling Technology | #54062 |
| P300 | Abcam | ab275379 |
| Flag tag | Cell Signaling Technology | #14793 |
| Myc tag | Cell Signaling Technology | #2272 |
| HA tag | Abcam | ab236632 |
| ACSL4 | Abcam | ab155282 |
| GPX4 | Cell Signaling Technology | #52455 |
| FTH1 | Abcam | ab75973 |
| COX2 | Abcam | ab179800 |
| CD4 | Abcam | ab288724 |
| CD8 | Abcam | ab217344 |
| CD19 | Cell Signaling Technology | #90176 |
| PD1 | Cell Signaling Technology | #84651 |
| Tim3 | Abcam | ab241322 |
| panCK | Abcam | Ab7753 |
| Epcam | Abcam | ab71916 |
| Zombie （BV510） | Biolegend | #423101 |
| CD45 （APC/CY7） | Biolegend | #157204 |
| CD4 （BV421） | Biolegend | #116008 |
| CD8 （Percp/CY5.5） | Biolegend | #100734 |
| CD19 （PE） | Biolegend | #115508 |
| CD11b （Percp/CY5.5） | Biolegend | #101228 |
| CD25 （APC） | Biolegend | #102012 |
| CD49b （APC） | Biolegend | #108910 |
| Ly6C （BV510） | Biolegend | #128033 |
| Ly6G （APC/CY7） | Biolegend | #127624 |
| IFNγ （APC/CY7） | Biolegend | #505849 |
| PD1 | Biolegend | #135216 |

| | | |
|---|---|---|
| （PE/CY7） | | |
| Tim3<br>（Percp/CY5.5） | Biolegend | #119717 |

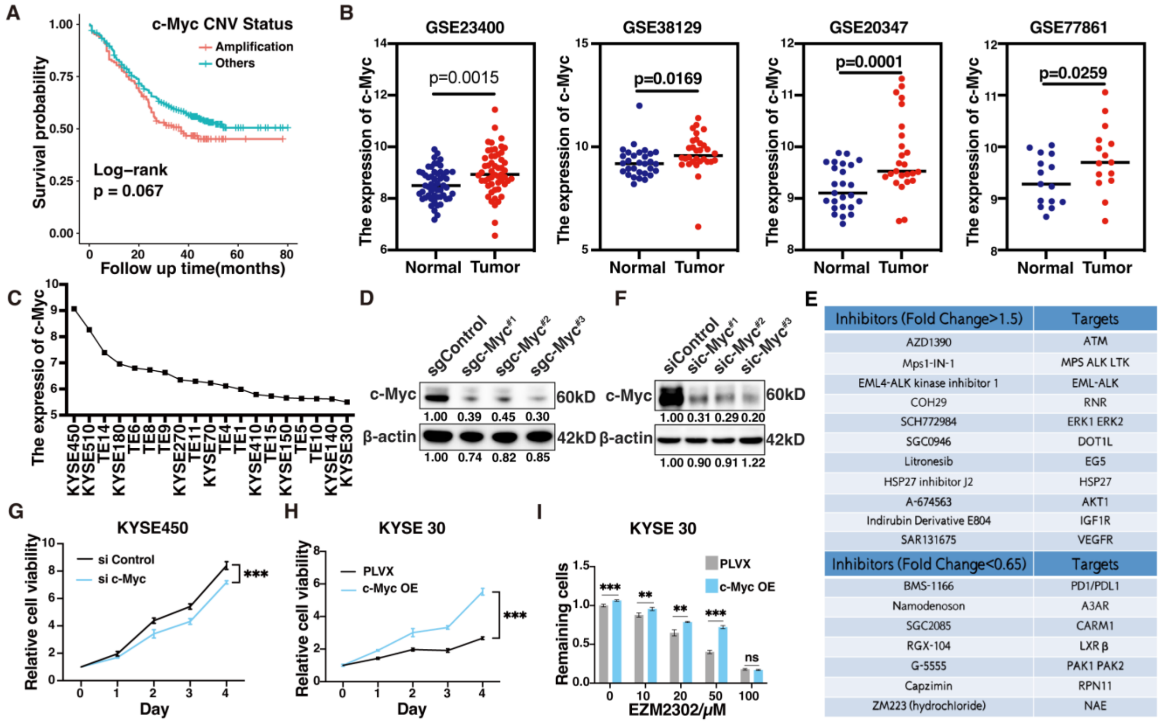


**Figure S1. Screening of drug combination regimens targeting *c-Myc***

(A) Kaplan-Meier survival curve showing that amplification of *c-Myc* correlated with lower disease-free survival in patients with ESCC (P=0.067). (B) The expression of *c-Myc* between normal tissue and tumor in GSE23400, GSE38129, GSE20347, and GSE77861. (C) The expression of *c-Myc* in esophageal cancer cell lines in the CCLE website. (D) Western blot analyses of the c-Myc level in control and c-Myc-knockout groups. (E) Inhibitors after the first round of screening. (F) Western blot analyses of the c-Myc level in control and si-c-Myc groups. (G) Growth curves were measured using CCK-8 to analyse KYSE450 cells stably transfected with control vector (black) or si c-Myc vector (blue) for 96 h. (H) Growth curves were measured using CCK-8 to analyse KYSE30 cells stably transfected with control vector (black) or *c-Myc* vector (blue) for 96 h. (I) Cell viability of PLVX or c-Myc-OE KYSE30 cells after treatment for 24 hours with EZM2302 in different concentration.

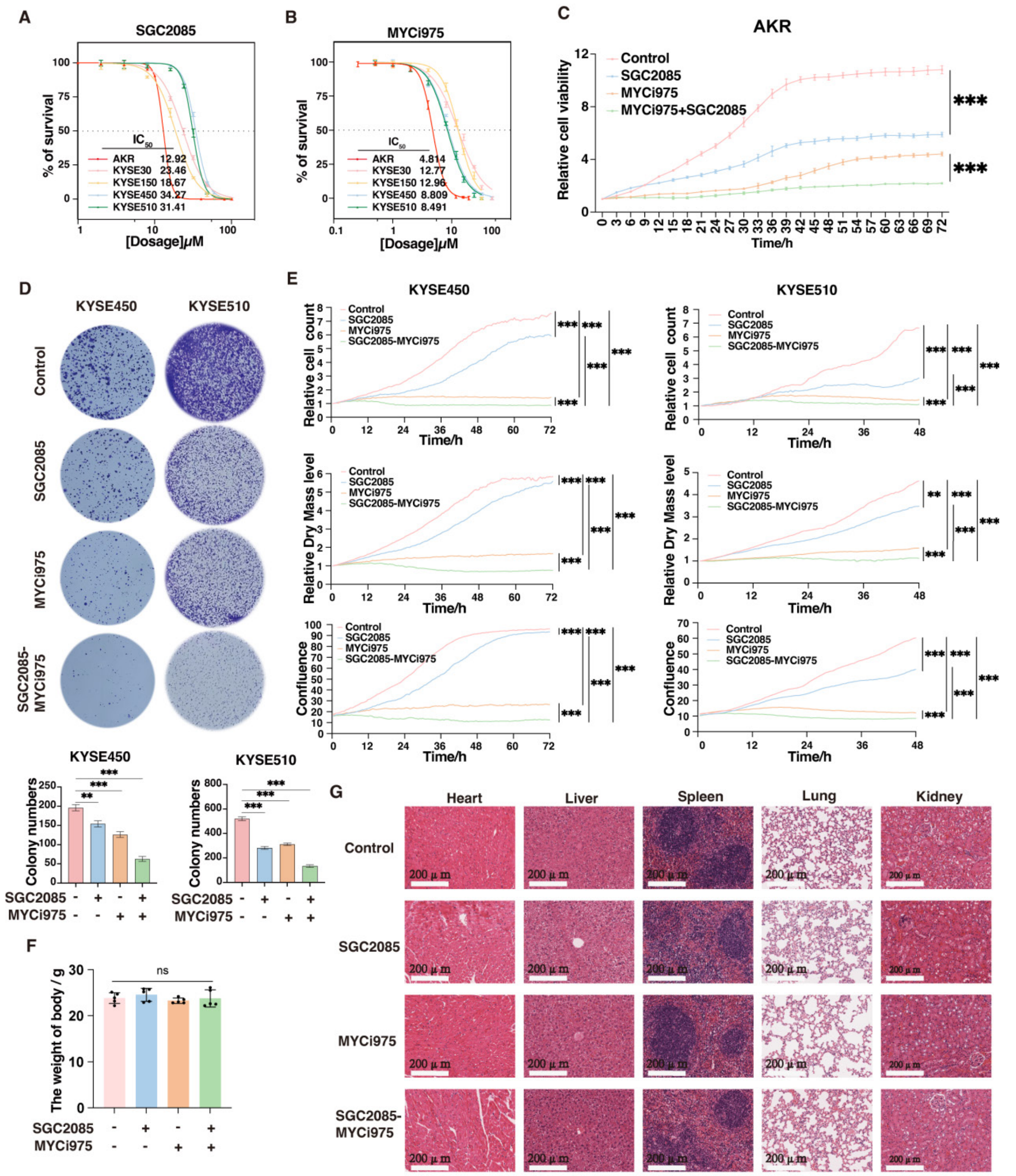


**Figure S2. Combination of *CARM1* and *c-Myc* inhibitor significantly inhibits esophageal squamous carcinoma cell proliferation**

(A-B) Inhibition curves (measured using CCK8 assays) of KYSE30, KYSE150, KYSE450, KYSE510 and AKR cells treated with SGC2085 (A) or MYCi975 (B) for 24 h. (C) Growth curves were measured using Incucyte live-cell analyses of AKR cells in control or medicated groups for 72 h. (D) Colony formation assay to detect and statistically analyze the proliferation of KYSE450 and

KYSE510 in control or medicated groups. (E) Growth curves, including cell count, dry mass and confluence were measured using Livecyte analyses of KYSE450 (left) or KYSE510 (right) cells in control or medicated groups for 48 h. (F) The weight of mice in animal experiments. (G) Pathologic findings in various organs after drug use.

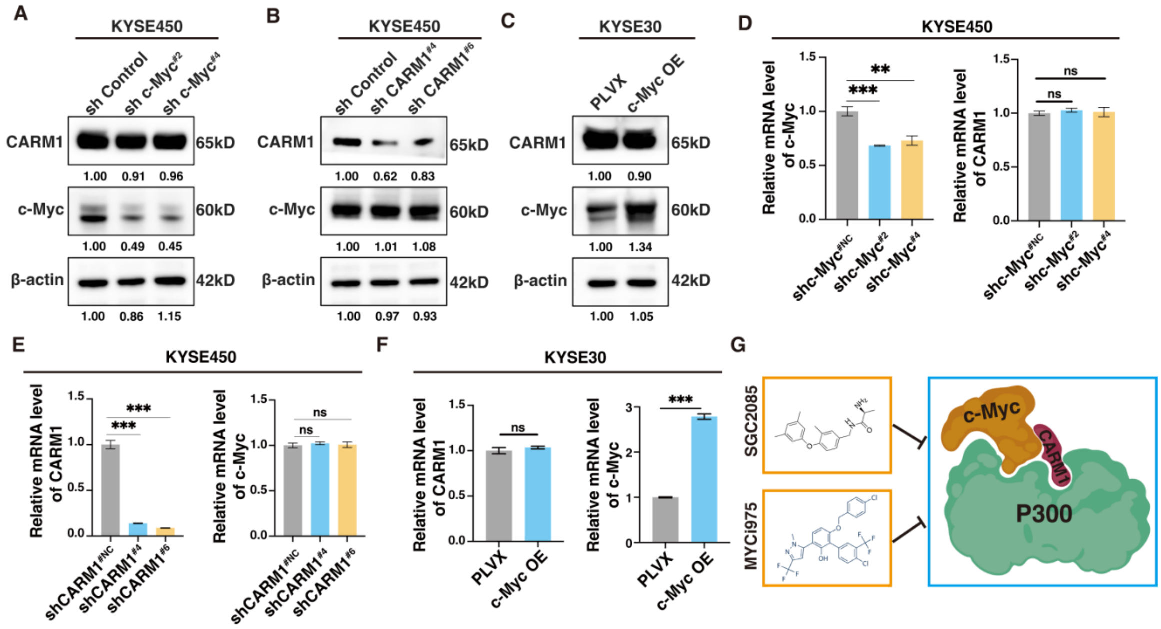

**Figure S3. CARM1 and c-Myc form a transcription complex with p300**

(A-C) Western blot analyses of the c-Myc and CARM1 levels in control and c-Myc-knockdown (A), CARM1-knockdown (B) and c-Myc-overexpressed (C) groups. (D-E) RT-qPCR analyses of the c-Myc and CARM1 levels in control and c-Myc-knockdown (D), CARM1-knockdown (E) and c-Myc-overexpressed (F) groups. Data were presented as mean±SD; n=3. Two-tailed t-tests. (G) Diagram of transcriptional complex CPC (c-Myc-p300-CARM1).

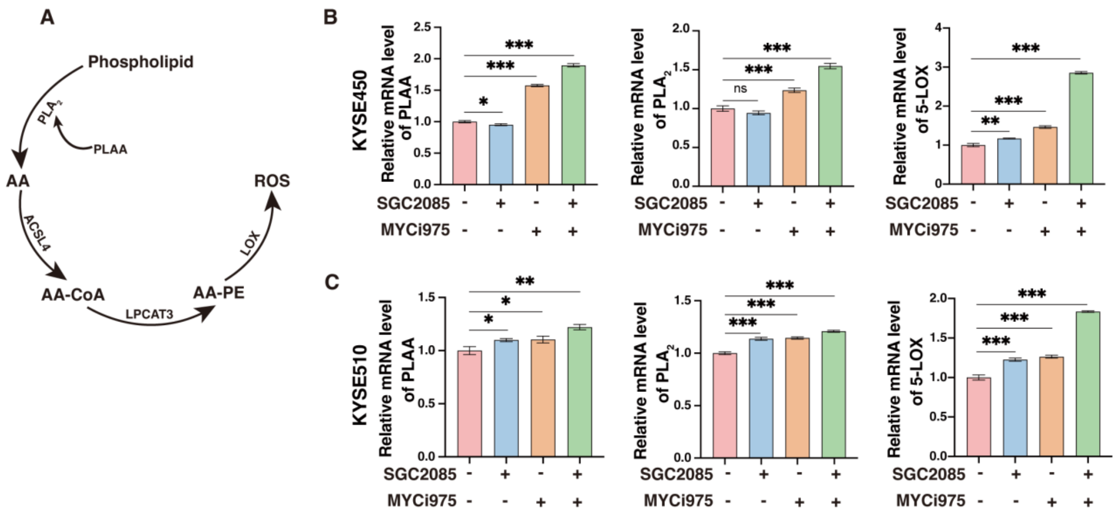


**Figure S4. Combination of *CARM1* and *c-Myc* inhibitor significantly upregulates arachidonic acid levels**

(A) Metabolic pathway of Arachidonic Acid. (B-C) RT-qPCR analyses of the *PLAA*, $PLA_2$ and *5-LOX* levels in control and medicated groups in KYSE450 (B) and KYSE510 (C) cells. Data were presented as mean±SD; n=3. Two-tailed t-tests.

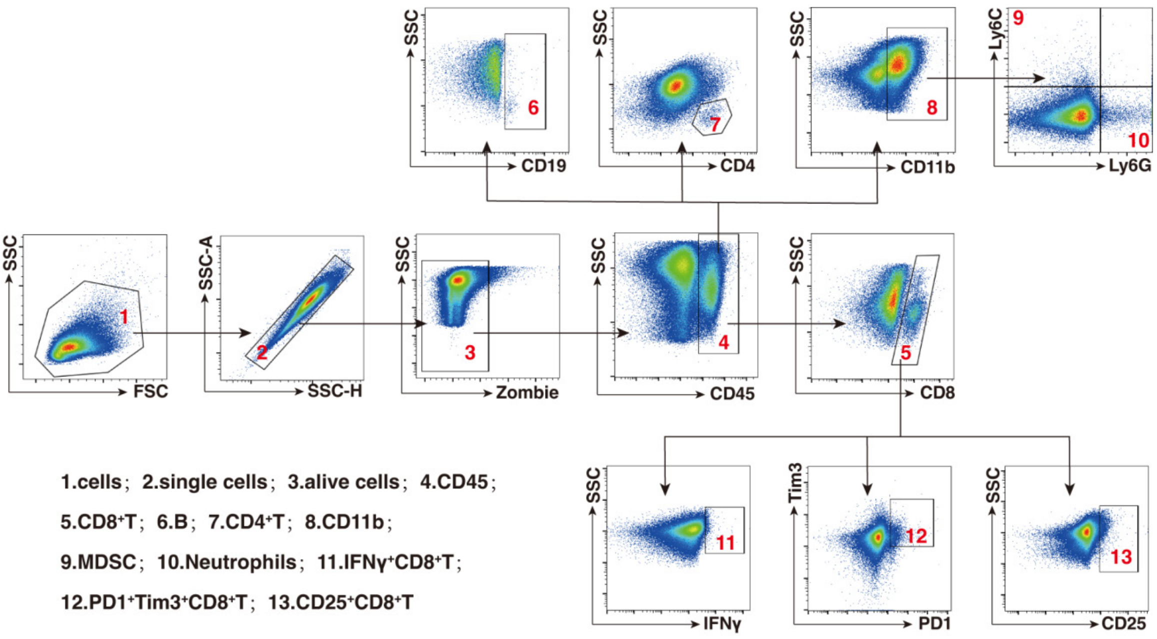


**Figure S5. Combination of *CARM1* and *c-Myc* inhibitor significantly enhances CD8$^{+}$ T cell infiltration within tumors**

Gating strategy for flow cytometry to identify distinct cell populations in mouse tumor.
